# Microglia Signaling Pathway Reporters Unveiled Manganese Activation of the Interferon/STAT1 Pathway and Its Mitigation by Flavonoids

**DOI:** 10.1007/s12035-023-03369-w

**Published:** 2023-05-04

**Authors:** Valeri V. Mossine, James K. Waters, Grace Y. Sun, Zezong Gu, Thomas P. Mawhinney

**Affiliations:** 1 Department of Biochemistry, University of Missouri, Columbia, MO 65211 USA; 2 Agriculture Experiment Station Chemical Laboratories, University of Missouri, Columbia, MO 65211 USA; 3 Department of Pathology and Anatomical Sciences, University of Missouri, Columbia, MO 65211 USA; 4 Department of Child Health, University of Missouri, Columbia, MO 65211 USA

**Keywords:** Manganese, *piggyBac* transposon, Interferon-sensitive response element ISRE, Apigenin, Myricetin, EGCG

## Abstract

Neuroinflammatory responses to neurotoxic manganese (Mn) in CNS have been associated with the Mn-induced Parkinson-like syndromes. However, the framework of molecular mechanisms contributing to manganism is still unclear. Using an in vitro neuroinflammation model based on the insulated signaling pathway reporter transposon constructs stably transfected into a murine BV-2 microglia line, we tested effects of manganese (II) together with a set of 12 metal salts on the transcriptional activities of the NF-κB, activator protein-1 (AP-1), signal transducer and activator of transcription 1 (STAT1), STAT1/STAT2, STAT3, Nrf2, and metal-responsive transcription factor-1 (MTF-1) via luciferase assay, while concatenated destabilized green fluorescent protein expression provided for simultaneous evaluation of cellular viability. This experiment revealed specific and strong responses to manganese (II) in reporters of the type I and type II interferon-induced signaling pathways, while weaker activation of the NF-κB in the microglia was detected upon treatment of cells with Mn(II) and Ba(II). There was a similarity between Mn(II) and interferon-γ in the temporal STAT1 activation profile and in their antagonism to bacterial LPS. Sixty-four natural and synthetic flavonoids differentially affected both cytotoxicity and the pro-inflammatory activity of Mn (II) in the microglia. Whereas flavan-3-ols, flavanones, flavones, and flavonols were cytoprotective, isoflavones enhanced the cytotoxicity of Mn(II). Furthermore, about half of the tested flavonoids at 10–50 μM could attenuate both basal and 100–200 μM Mn(II)-induced activity at the gamma-interferon activated DNA sequence (GAS) in the cells, suggesting no critical roles for the metal chelation or antioxidant activity in the protective potential of flavonoids against manganese in microglia. In summary, results of the study identified Mn as a specific elicitor of the interferon-dependent pathways that can be mitigated by dietary polyphenols.

**Supplementary Information:**

The online version contains supplementary material available at 10.1007/s12035-023-03369-w.

## Introduction

Numerous observational and experimental studies have established an association between environmental pollutants and elevated risk of various neurological disorders in humans. To this end, overexposure to or dysregulation of metals in CNS has been implicated in Alzheimer’s, Parkinson’s, and Wilson’s diseases [1, 2]. One of such metals is manganese, an important bioelement, which is also extensively used by humans, especially as a component of stainless-steel alloys. The Parkinson disease-like symptoms, which appear as a result of excessive manganese accumulation in the brain and which could not be ameliorated by dopamine precursor levodopa [3], had been recorded for many years in workers exposed to Mn-containing aerosols at steel mills, mines, or welding [4] and are currently known as manganism symptoms. However, molecular and cellular mechanisms of the manganese neurotoxicity are not yet fully understood. A limited number of agents have been proposed to attenuate neurotoxicity of manganese, including polyphenols silymarin [5], resveratrol [6], curcumin [7], and quercetin [8], metal chelators EDTA [9], *p*-aminosalicylic acid [10, 11], and antioxidant RibCys [12]. Nonetheless, no effective therapy currently exists to treat manganese poisoning in the brain.

Microglia are macrophage-like cells residing in the CNS. These cells are charged with a variety of brain defense and homeostasis tasks, from scavenging infected and damaged cells or cell debris to regulating regeneration and remodeling of the brain neuronal networks [13]. Transformation of microglia to reactive states in response to pathological stimuli, or microglial activation, can also be neurotoxic and contribute to the neurodegenerative processes in the brain [14]. Thus, it has been proposed that cellular mechanisms of Mn neurotoxicity may involve, among others, disruption of function and chronic inflammatory activation of microglia and astrocytes [15]. Suggested molecular mechanisms of Mn cytotoxicity implicate oxidative stress, impairment of glutaminase activity, and transcriptional and functional deregulation of the glutamate transporters in glial cells [16, 17]. The suggested pathways involved in Mn-induced inflammatory activation of glial cells have been limited, so far, to the signaling cascades of NF-κB [15, 18] and JAK2/STAT3 [19] pathways plus subsequent production of pro-inflammatory inducible nitric oxide synthase (iNOS), cyclooxygenase-2 (COX-2), interferon-alpha, interferon-beta, and interferon-gamma (IFN-α/β/γ), tumor necrosis factor (TNFα), interleukin-1β and interleukin-6 (IL-1β, IL-6) [20, 21]. In addition, Sengupta et al. [22] reported a gene expression profile in manganese chloride-treated primary human astrocytes and, according to this report, out of 20 targets positively affected by the IFN-γ/JAK/STAT1 pathway, sixteen were upregulated by Mn. Based on these findings, it could be hypothesized that pro-inflammatory signaling stimulated by Mn in microglia may include the IFN-γ pathway, as well.

Signal transducer and activator of transcription 1 (STAT1) is a cytoplasmic inducible transcription factor which relays extracellular signals, typically provided by type I (IFN-α, β, δ, etc.), type II (IFN-γ), or type III (IFN-λ) interferons and certain cytokines [23, 24]. Two principal signaling pathways leading to activation of STAT1 involve recognition of the interferons by, respectively, type I/type III or type II interferon receptors, a subsequent activation of associated Janus tyrosine kinases (JAKs), and phosphorylation of cytoplasmic STAT1 by JAK. Activated STAT1 then forms either homodimer or heterodimer with STAT2. The homodimer STAT1 is directly translocated to the nucleus, where it regulates gene expression at the gamma-interferon activated DNA site (GAS). The heterodimer STAT1/2 forms a complex with Interferon Regulatory Factor 9 (IRF9) and then binds to DNA, predominantly at a different gene regulatory sequence called interferon-stimulated response element (ISRE). When bound to the GAS, the STAT1 can upregulate production of a number of pro-inflammatory enzymes and mediators, including NOS2, COX-2, TNF-α, IL-1β, IL-6, IL-12, IL-23, MCP-1, or IFN-γ [25, 26]. As a consequence, IFN activation of STAT1 in microglia and peripheral macrophages may promote their transformation into the pro-inflammatory phenotypes [27, 28].

Recently, we have reported a successful application of insulated reporter transposons in astrocytes for assessment of activity of multiple neuroinflammation-related transcription factors, including the STAT1 [29]. In this paper, we describe testing of the reporter construct in BV-2 microglia cell line, by taking on an objective of searching for potential pro-inflammatory metals and their inhibitors in microglia. Here, we report for the first time activation of the gamma-interferon-dependent JAK/STAT1 pathway in Mn-treated microglial cells. Next, we describe the effects of 64 flavonoids on the transcription factors implicated in neuroinflammation, namely STAT1, STAT1/2, STAT3, NF-κB, AP-1, and Nrf2. Finally, we elucidate the preventive potential of the flavonoids against manganese-induced transcriptional activation of the homodimer STAT1.

## Materials and Methods

### Chemicals

A collection of metal salts, biochemicals, and flavonoids was from various reagent vendors (Supplementary Table S1). The compounds were of reagent grade (> 90%) and used without further purification. Stock solutions of flavonoids were prepared in DMSO/propylene glycol (1:3 v/v) at 20 mM concentrations and were stored at − 20 °C until use. Lipopolysaccharide (LPS) from *Pseudomonas aeruginosa* was purchased from MilliporeSigma.

### Cell Culture

Immortalized murine microglia cell line BV-2 [30] was provided by Dr. Grace Sun (University of Missouri) who received an original batch from Dr. Rosario Donato (University of Perugia, Italy). The cells, as well as the BV2-based reporter transfects, have been routinely cultured in 1:1 DMEM/F12 Ham media mixture (MilliporeSigma) supplemented with 5% newborn calf serum (NCS, HyClone) and 1% (v/v) penicillin/streptomycin cocktail (pen/strep, HyClone). This medium is henceforth referred to as the DMEM/F12 complete medium. To passage, the cells were treated with 0.05% trypsin (MP Biochemicals) in 1:1 Corning CellStripper cocktail/PBS (MilliporeSigma) and subcultured at 1:5 ratio upon reaching 60–70% confluency. The standard culturing conditions for all cells were 37 °C, 5% CO_2_, and 100% humidity.

### Plasmid Constructs

Super *piggyBac* transposase expression vector was purchased from System Biosciences. Vectors *p*TR01F, *p*TR05F, *p*TR09F, *p*TR13F, *p*TR23F, and *p*TR34F have been previously reported [29, 31, 32]. *p*TR25F vector has been assembled as follows: inserts containing 4-bp overhang sequences for the ligation reaction and total of 8 interferon-sensitive response elements (ISRE) for binding the heterodimer transcription factor STAT1/STAT2 (Table 1) were synthesized and annealed. The inserts and the larger product of *p*TR01F [31] digestion with the *Nhe*I/*Bgl*II were uniformly assembled into *p*TR25F in one ligation step. Correctness of the insertion was confirmed by DNA sequencing.

**Table 1:** Octamer interferon-sensitive response element sequences for the construction of *p*TR25F vector

Insert	Sequence
Sense	ctagcTAGTTTCACTTTCCCTAGTTTCACTTTCCCTAGTTTCACTTTCCCTAGTTTCACTTTCCCTAGTTTCACTTTCCCTAGTTTCACTTTCCCTAGTTTCACTTTCCCTAGTTTCACTTTCCCa
Antisense	gatctGGGAAAGTGAAACTAGGGAAAGTGAAACTAGGGAAAGTGAAACTAGGGAAAGTGAAACTAGGGAAAGTGAAACTAGGGAAAGTGAAACTAGGGAAAGTGAAACTAGGGAAAGTGAAACTAg

### Stable Transfections

To generate stable reporter BV2.RnnF lines, the original BV-2 cells were seeded into wells of a 96-well plate, at 5 × 10^4^ cells per well in complete DMEM/F12 medium and left to adhere for 6 h. The cells were then treated with the NATE inhibitor cocktail (InvivoGen) for 30 min, followed by a mixture of 100 ng *p*TRnnF reporter plasmids and 33 ng Super *piggyBac* transposase plasmid complexed with Lipofectamine 2000 transfection reagent (Invitrogen) at 1:2 (μg DNA/ μL reagent) ratios. After 16 h, regular media were added and cells were left to proliferate for the next 48–72 h. The transfected cells were then treated with the selecting antibiotic (5 μg/mL puromycin) for another week, and the surviving cells were expanded for cryopreservation and activity validation. When the numbers of reporter cells were expanded for experiments, the complete DMEM/F12 medium was typically supplemented with 2 μg/mL puromycin.

### Cell Treatment Schedule

Typically, original BV-2 or reporter BV2.RnnF cells were plated in Nunclon Delta Edge 96-well plates (ThermoFisher) at 1 × 10^4^ cells/well in 100 μL of an adaptation low-serum medium consisted of the DMEM/F12 media mixture supplemented with 2 mg/L insulin, 2 mg/L transferrin, 2 μg/L selenite (2-ITS), 2% NCS, and the pen/strep antibiotic. After 40 h, the adaptation medium was replaced with the Phenol Red-free 5:5:1 DMEM/F12/RPMI-1640 mixture, supplemented with 1 g/L BSA, the 2-ITS mix and the pen/strep (the test medium). The cells were cultured for next 6 h, after which time, the medium was replaced with fresh test medium containing 0.5% of the DMSO/propylene glycol mix (carrier solvent controls), metal salts, flavonoids, and/or specific inducer agents, and the plates were incubated for the next 18 h or other indicated times.

### Reporter Activity Assay

In a typical experiment, immediately after the treatments, the reporter cells in 96-well plates were carefully washed with PBS and lysed in 70 μL of the lysing buffer [29] for 16 h at 8 °C. The GFP fluorescence values in the lysates were measured at the 482(9)/512(17) nm wavelength (slit width) setup and was followed by an addition of 20 μL luciferase substrate [29]. Kinetic luminescence readings in the wells were done in 2-min intervals for 8 min total. All the measurements were done using a Synergy MX (BioTek) plate reader. The GFP fluorescence values were used for both evaluation of relative cell transcriptional activity/proliferation and normalization of the reporter luciferase activities in respective wells.

### Plotting and Statistical Analysis

Statistical tests and plots were done using SigmaPlot, version 13.0. Datasets are presented as mean ± SD. Individual data points are plotted where applicable.

## Results

### Validation of BV-2 Reporters

**Fig. 1:**
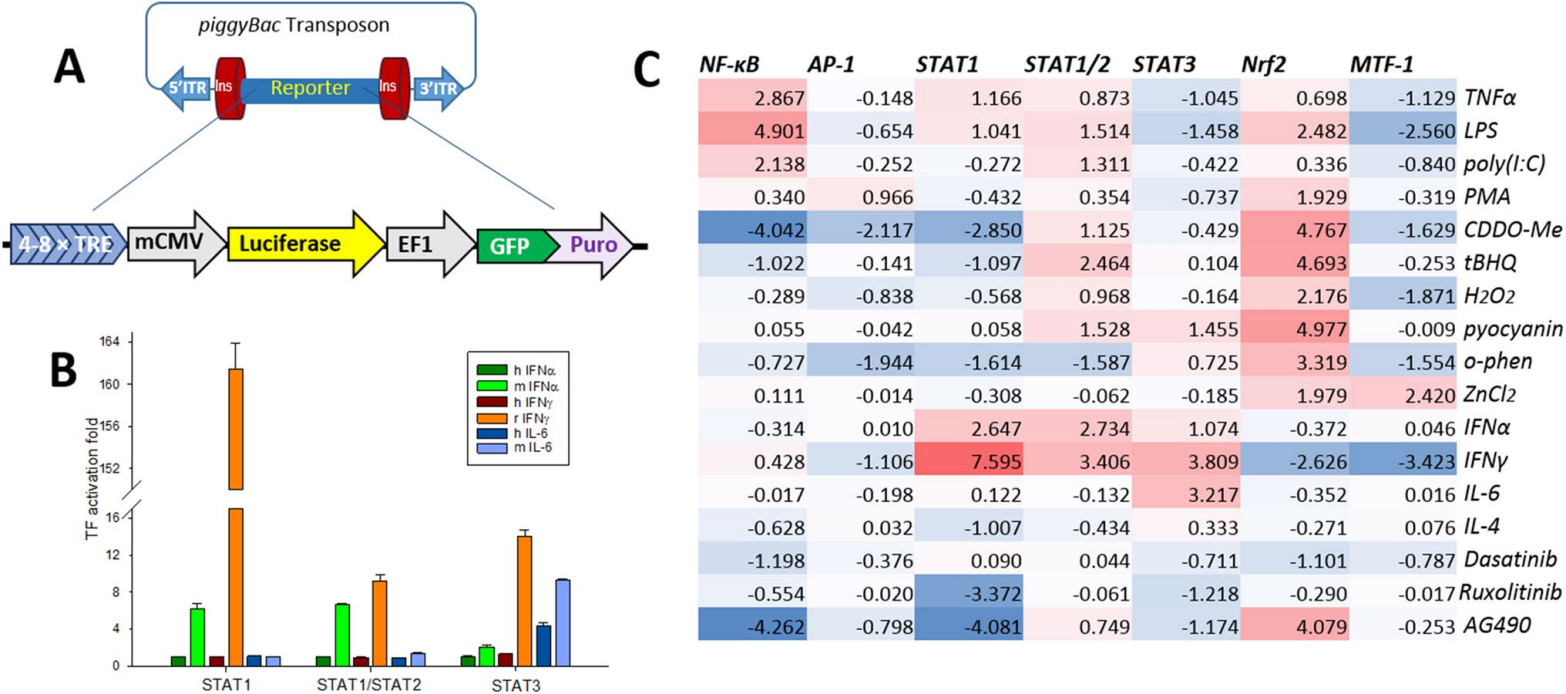
Establishing transcriptional activation assay in immortalized murine microglia BV-2. **A** A general scheme of the reporter construct. Four to eight specific transcription factor binding sequences (transcription factor response elements, TREs) and the mCMV promoter regulate reporter firefly luciferase, while the EF1 promoter provides constant production of destabilized copepod GFP and puromycin resistance selector. The flanking insulators protect from epigenetic silencing of the reporter, while the *piggyBac* transposon ITRs secure accurate and efficient insertion of the reporter into the genomic DNA. **B** Comparative TF activity in reporter cells treated with human vs rodent pro-inflammatory cytokines. Concentrations of human and murine cytokines, 20 ng/mL; rat interferon-γ, 2 ng/mL. The error bars are SDs for *n* = 3. **C**. Heatmap of the reporter activation, expressed as log_2_(TF induction fold), to common inducers and inhibitors used to validate specificity of the reporters. See extended Supplementary Table S2 for viabilities and SDs. Concentrations: tumor necrosis factor (TNFα), 5 ng/mL; lipopolysaccharide (LPS), 100 ng/mL; poly(I:C), 5 μg/mL; phorbol 12-myristate 13-acetate (PMA), 100 ng/mL; Bardoxolone (CDDO-Me), 500 nM; *tert*-butyl hydroquinone (tBHQ), 10 μM; hydrogen peroxide, 250 μM; pyocyanin, 60 μM; *o*-phenanthroline, 20 μM; ZnCl_2_, 20 μM; mouse interferon-α (m IFNα), 10 ng/mL; rat interferon-γ (r IFNγ), 5 ng/mL; mouse interleukin-6 (m IL-6), 10 ng/mL; mouse interleukin-4 (IL-4), 25 ng/mL; Dasatinib, 200 nM; Ruxolitinib, 50 nM; Tyrphostin B42 (AG490), 50 μM

A general scheme of the reporter constructs used in this study is shown in Fig. 1A. We have developed a unique set of insulated, *piggyBac* transposable reporters consisting of a specific transcription factor response element to regulate expression of the firefly luciferase gene. The elongation factor 1 (EF1) promoter for continuous activation of the copepod GFP gene fused with a puromycin resistance sequence allows for simultaneous evaluation of cellular viability, normalization of the luciferase activity, and selection of cells with the reporter resistant to epigenetic silencing. In addition to previously reported plasmids [29, 31, 32], we have assembled a novel reporter vector that carries eight binding sites, ISRE sequences, for the heterodimer transcription factor STAT1/STAT2. The BV-2 microglia were stably transfected with this plasmid, as well as with the reporters for transcriptional activity of the NF-κB, AP-1, homodimer STAT1, STAT3, Nrf2, and MTF-1. The new reporter cell lines were tested for selectivity (Fig. 1, Supplementary Table S2). As expected, activity of the NF-κB, AP-1, STAT1, STAT1/STAT2, STAT3, and MTF-1 increased upon treatment of the microglia with respective specific inducers, namely LPS, PMA, IFN-γ, IFNα, IL-6, CDDO-Me, and ZnCl_2_. Microglia were particularly sensitive to IFN-γ, which induced over hundredfold increase in the STAT1 activity. Interestingly, transcriptional activity of the STAT1/STAT2, but not the NF-κB, AP-1 and STAT1, significantly increased upon treatment of the microglia with redox stressors and classical inducers of Nrf2 such as bardoxolone, *tert*-butyl hydroquinoline, hydrogen peroxide, and pyocyanin [32]. Among other surprising responses were high inhibitory activity of IFN-γ against MTF-1 and the anti-inflammatory effects of an iron chelator *o*-phenanthroline. Murine BV-2 microglia did not recognize human IFN-α and IFN-γ but was species-tolerant towards IL-6 (Fig. 1B).

### Manganese Uniquely Induces Transcriptional Activities of the NF-κB, STAT1, and STAT1/2

To explore the pro-inflammatory responses in the newly developed set of BV-2 microglia-based reporters, we exposed the cultured microglia to 13 metal salts, which are of common interest to the neurotoxicology field. This initial testing revealed surprisingly strong responses in manganese-treated reporters for transcriptional activity of the homodimer STAT1 and heterodimer STAT1/STAT2 (Table 2). In addition to these interferon-dependent signaling pathways, manganese also induced activation of the pro-inflammatory NF-κB and JAK/STAT3 pathways, although to a much smaller effect. Among the rest of tested metal salts, only 1 mM barium chloride promoted activation of the NF-κB to a comparable extent. Barium did also promote activation of the oxidative/electrophilic stress sensor KEAP-1/Nrf2, as did other established Nrf2 activators Cd(II), Co(II), Fe(III), Pb(II), V(V), and Zn(II), at tested metal concentrations. Notably, manganese (II) had no effect on the transcription factor Nrf2 but exerted a strong inhibition of the MAPK/JNK/AP-1 and zinc-sensory MTF-1 pathways.

**Table 2:**
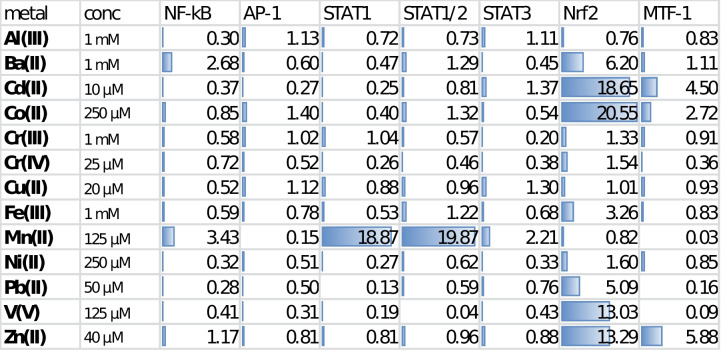
Activation folds of seven transcription factors in BV-2 microglia exposed to metal salts for 14 hours. See Supplementary Figures S1 –S7 for extended viability and dose-response data

**Fig. 2:**
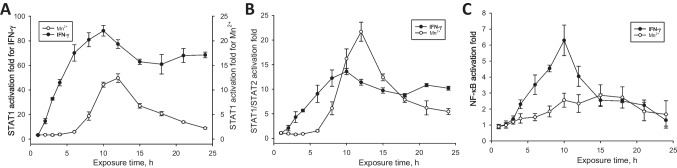
Time course of transcriptional activation of the **A** homodimer STAT1, **B** heterodimer STAT1/STAT2, and **C** NF-κB transcription factors in BV-2 microglia exposed to 2 ng/mL IFN-γ or 100 μM manganese(II) citrate. The experimental curves in **A** have been drawn to different scales, for clarity

Next, we have assessed a comparative temporal activation of the interferon-dependent and the NF-κB signaling pathways in BV-2 microglia by manganese (II) and interferon-γ. As illustrated in Fig. 2, both IFNγ- and Mn(II)-induced activation of the pathways proceeded through maxima, which, in the IFNγ-treated reporter cells, were achieved in about 10 h. The activation of microglia treated with Mn(II) lagged until about 6 h, then reached maxima at about 12 and 15 h in the reporters of the interferon-dependent and the NF-κB signaling pathways, respectively.

We further asked whether Mn(II) could interact with specific pro-inflammatory activators at the ISRE and GAS in microglia. A number of previous studies have demonstrated the ability of Mn to potentiate effects of the NF-κB activators, such as LPS, TNFα, or IFNγ [20, 33]. Accordingly, we tested combinations of manganese (II) with IFNα, IFNγ, LPS, poly(I:C), and β-amyloid peptide (25-35) in the reporters of STAT1 and STAT1/2 transcriptional activation in microglia. There were no or weak interactions between Mn(II) and the interferons, poly(I:C), and Aβ_25-35_ (Supplementary Tables S3–S7). However, the Mn(II)/LPS combinations revealed a trend of the antagonism at increasing concentrations of these agents (Table 3). The same trend was observed for the IFNγ/LPS combinations (Table 3).

**Table 3:**
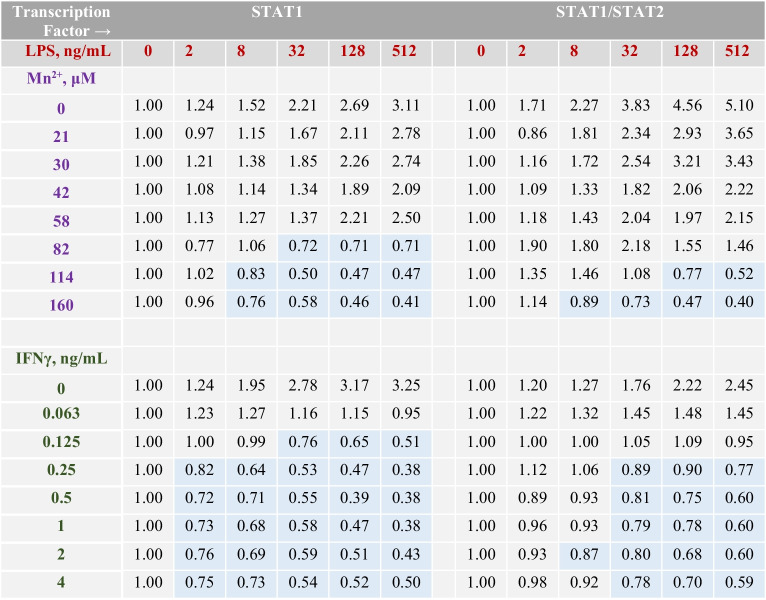
Relative TF activation folds in BV-2 microglia treated with combinations of LPS and Mn(II) or combinations of LPS and IFNγ for 12 h, the data points are single measurements. In each row, the activation folds are normalized to Mn(II)-only treated cells at indicated Mn(II) concentrations (Supplementary Table S3). Data highlighted in blue indicate antagonistic interactions between Mn(II) and LPS or between IFN-γ and LPS in microglia

### Flavonoids Differentially Affect Basal Activities of the Neuroinflammation-Related Transcription Factors NF-κB, AP-1, STAT1, STAT1/STAT2, STAT3, and Nrf2

**Fig. 3:**
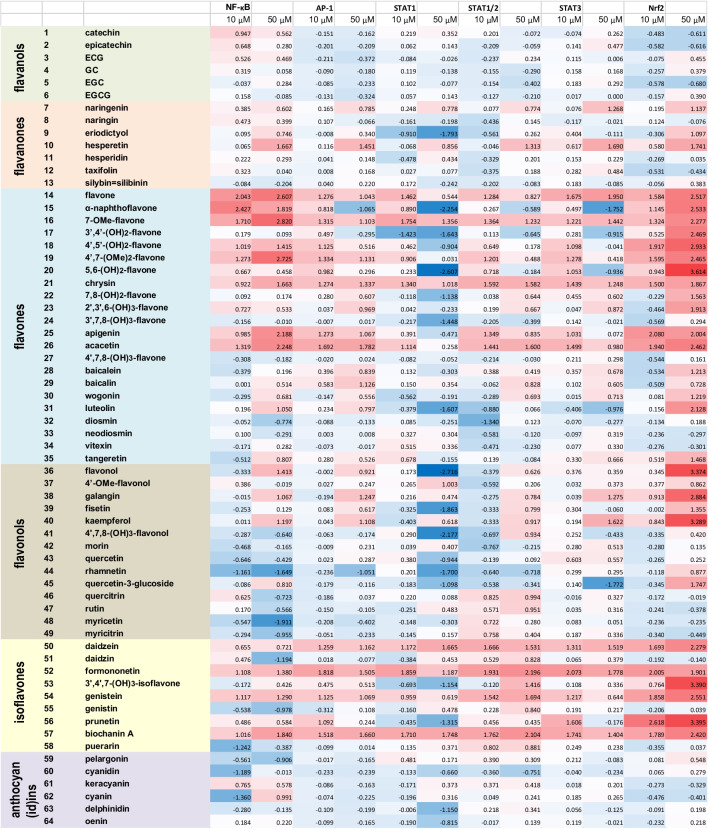
Responses of neuroinflammation-related signaling pathways to treatments of BV-2 microglia reporter cells with flavonoids at 10 μM and 50 μM concentrations. Heatmap of the reporter activation expressed as log_2_(TF induction fold). See extended Supplementary Table S8 for cell viabilities and SDs

In order to demonstrate the utility of our microglia-based reporters for high-throughput protocols, we performed evaluation of multiple natural and synthetic flavonoids as potential inducers/inhibitors of signaling pathways implicated in neuroinflammation. Specifically, in a single experiment, we tested 64 flavonoids (Supplementary Table S1), at two concentrations, 10 μM and 50 μM, 3 samples per agent dose, in 6 reporter lines. Together with the assay controls, about 2500 biological samples were processed in one run, yielding about 5000 data points, given that each biological sample provided a cell lysate for the GFP fluorescence and luciferase luminescence readouts. The results of the experiment are summarized in Fig. 3 and Supplementary Table S8. There is a significant variability in the reporter responses to individual flavonoids, but certain patterns can be seen in Fig. 3. Thus, structural classes of flavan-3-ols and anthocyan(id)ins generally suppress the basal levels of all six transcriptional factors, while flavones and isoflavones induce an increase in activities of these TFs. Several individual flavonoids, including flavone, chrysin, acacetin, daidzein, formononetin, genistein, and biochanin A, acted as “pan-activators” of all six signaling pathways in the reporter microglia. Among the reporters, STAT1 was the most sensitive to inhibition by flavonoids, while the redox sensor Nrf2 was activated by the majority of tested flavonoids at 50 μM.

### Effects of Flavonoids on Manganese Cytotoxicity and Manganese-Induced Transcriptional Activation of the Homodimer STAT1

**Fig. 4:**
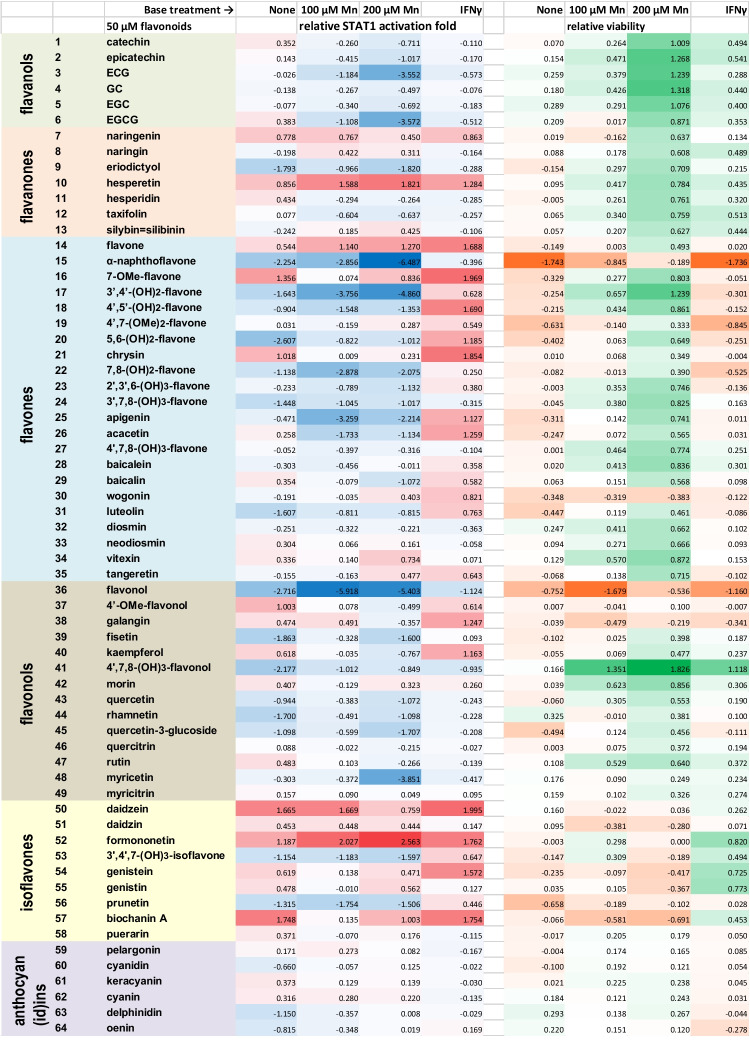
Responses of neuroinflammation-related signaling pathways to treatments of BV-2 microglia reporter cells with combinations of 50 μM flavonoids and manganese(II) at 100 μM or 200 μM, or 50 μM flavonoids and interferon-γ at 1 ng/mL for 12 h. Heatmap of the reporter activation expressed as log_2_(relative TF induction fold) and relative viability expressed as log_2_(relative GFP expression fold). The relative TF induction and GFP expression folds in columns are calculated as the luciferase activity or GFP fluorescence in the combination-treated cells divided by the luciferase activity/GFP fluorescence in reporter cells subjected to the respective base treatment. See Supplementary Table S9 for the original data source

Having found that certain flavonoids can significantly inhibit basal activity of the type II interferon signaling pathway in BV-2 microglia, we also examined whether flavonoids could inhibit the manganese-promoted activation of this pathway. Cells were treated with combinations of 100 μM or 200 μM manganese(II) and 50 μM flavonoid for 12 hours. The chosen Mn(II) concentrations were close to pathophysiological levels of manganese (60–160 μM) found in the human brain [34]. In addition, this experiment included treating microglia with combinations of the 50 μM flavonoids with interferon-γ at 1 ng/mL. The results are given in Supplementary Table S9 and are illustrated in Fig. 4. Analysis of Fig. 4 reveals an overall trend of flavonoids to improve viability of Mn(II)-treated microglia. Exceptions are isoflavones as a group, cytotoxic α-naphthoflavone (#15) and flavonol (#36), as well as galangin (#38) and wogonin (#30). Interestingly, isoflavones could increase the viability of IFNγ-treated microglia, while 3,7,8,4′-tetrahydroxyflavone (#41) was the most effective in rescuing the microglia from Mn(II) cytotoxicity. Flavan-3-ols (catechins), flavones, and flavonols attenuated Mn(II)-induced STAT1 activation in microglia, with α-naphthoflavone and flavonol acting as the most potent inhibitors. Other phenolics, including epicatechin gallate (#3), EGCG (#6), 3′,4′-dihydroxyflavone (#17), and myricetin (#48), could counteract the pro-inflammatory activity of manganese (II) in BV-2 microglia, as well. On the other hand, several flavonoids co-operated with Mn(II) in stimulation of the STAT1; the most active were hesperetin (#10), flavone (#14), daidzein (#50), and formononetin (#52). Overall, the majority of tested flavonoids were poorly counteracting the pro-inflammatory action of IFNγ and, in fact, about one half in the list did enhance IFNγ-induced transcriptional activation of the STAT1 in BV-2 cells.

**Fig. 5:**
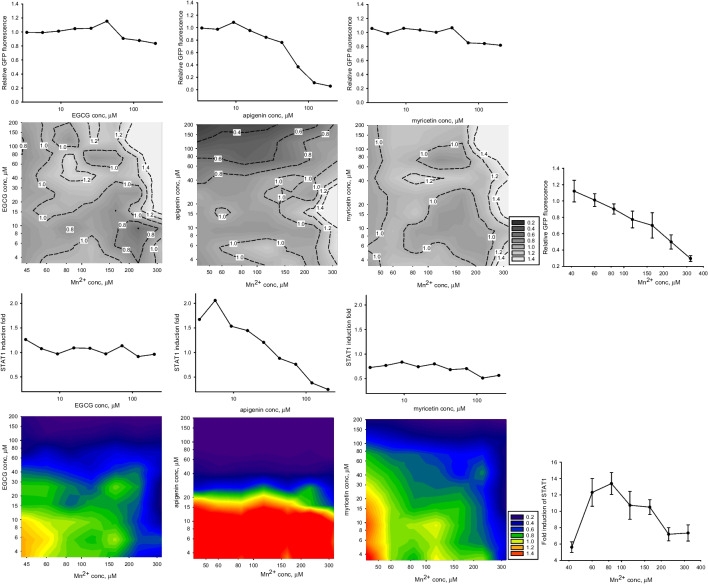
Viability (two upper rows) and transcriptional activation of homodimer STAT1 (two lower rows) in BV-2 microglia treated with manganese(II) and flavonoids EGCG, apigenin, and myricetin in combinations (contour plots) and alone (satellite line plots) for 12 h. The data points are single measurements or, wherever SD error bars are shown, *n* = 3. For contour plots, relative viability and relative STAT1 activation folds are calculated as in Table 3

One notable pattern seen in Fig. 4 is that the attenuation effects exerted by flavonoids on manganese-treated microglia were more prominent at 200 μM Mn(II) concentrations, as compared to 100 μM Mn(II). To investigate this unexpected effect at other concentrations, we selected three flavonoids, specifically EGCG, apigenin (#25), and myricetin. Unlike EGCG and myricetin, the attenuating effect of apigenin on STAT1 activation decreased in cells treated with 200 μM Mn(II), as compared to those treated with 100 μM Mn(II) (Fig. 4). The combination experiment yielded data presented as contour plots in Fig. 5. Several features can be noted when inspecting Fig. 5: (a) decrease in the STAT1 activation positively correlates with decrease in viability in microglia treated with Mn(II) or apigenin alone; (b) relative to Mn(II)-only treatment, viability of cells treated with Mn(II)/flavonoid combinations generally increased with increasing Mn(II) concentration, even in the case of cytotoxic apigenin; (c) in the Mn(II)/EGCG and Mn(II)/myricetin combinations, increase in concentrations of any agent could lead to inhibition of the relative STAT1 activity; (d) apigenin at concentrations below 15 μM could synergize with Mn(II) in the STAT1 activation.

## Discussion

An initial goal in this study was to develop a set of neuroinflammation-relevant signaling pathway reporters in a microglia cell line. Our previous experience with integration of the insulated reporter transposons into genomic DNA of several cell types, including an immortalized astrocyte, resulted in generation of multiple reporter cell lines, which demonstrated high consistency of the reporter response over prolonged cell culturing times [29, 31, 35]. The BV-2 microglia, however, has distinguished itself from other cell types by relatively low stability of the reporter activity, in a sense that continuous culturing of these cells without selecting antibiotic puromycin would often lead to decreased GFP and luciferase expression. Nevertheless, careful control of the reporter activity when expanding the cell numbers makes the BV-2 microglia reporters suitable for medium- to high-scale screening studies, as demonstrated in this work.

Our finding that manganese(II) can act as a strong inducer of the interferon-dependent signaling pathways in microglia is novel, and it concurs with the well-documented pro-inflammatory activities of this metal in CNS [15]. The molecular mechanisms of the manganese-induced neuroinflammation were typically explained by the cytotoxicity of this metal [36, 37]. Consequently, the main focus in such studies was on the activities and products of the stress-sensing transcription factors, such as NF-κB, AP-1, and YY1 [15, 18, 38–41]. In one study [22], a gene expression profile in Mn-treated astrocytes showed similarity with that targeted by interferon-γ, which prompted the authors to suggest that manganese could activate the interferon signaling pathway, even though no increase in the interferon transcription has been detected.

A few in vivo and in vitro studies with peripheral macrophages and other types of immune cells have recognized Mn(II) as an activator of the immune function through induction of type I interferon [42–44]. A proposed mechanism of manganese-stimulated production of type I interferons includes Mn(II) involvement in the cGAS-STING pathway and thus suggests a particular physiological function for manganese in innate anti-viral defense [43]. Taking into account our observations of the lag induction time (Fig. 2) and similarity in activation folds of both homodimer STAT1 and heterodimer STAT1/STAT2 by manganese(II) and IFNα (Fig. 1, Table 2), it would be tempting to ascribe such a role for Mn(II) in microglia, as well. However, a number of other observations obtained in this work cannot be explained based on current models of the manganese-induced macrophage activation. For instance, transcription of type I interferons downstream the cGAS-STING pathway is thought to proceed via the activation of NF-κB [43]. Conversely, in our experiments, the NF-κB activation has lagged well behind the STAT1 and STAT1/STAT2 activation profiles by Mn (Fig. 2). Activation of other transcription factors downstream of the cGAS-STING pathway, such as the interferon regulatory factor IRF3 and STAT6 [45], could be involved in type I interferon expression, as well. Yet, release of IFN-α/β by Mn-stimulated macrophages occurred after about 20-24 hours and reached its maximum at 48-72 hours post treatment [43, 46], while in our experiments, the maximal transcriptional responses of both the homodimer STAT1 and the heterodimer STAT1/2 occurred after only 12 h posttreatment with Mn. Finally, in all aforementioned literature experiments, manganese(II) acted rather as a potentiator than a primary inducer of the interferon production, by co-stimulating macrophages in combinations with viral RNA or DNA, in accord with the cGAS-STING pathway model.

Activation of two other transcription factors, NF-κB and STAT3, by manganese (II) in BV-2 microglia is well in accord with previous reports [19, 39]. The inhibitory effect of this metal on transcriptional activation of the MTF-1 could be related to a competition between Mn^2+^ and Zn^2+^ for their common metal transporters, such as ZIP8, ZIP14, and DMT-1 [47]. On the other hand, the inhibitory effect of Mn(II) on the MAPK/JNK/AP-1 pathway observed by us in BV-2 microglia is in a contrast with data reported by Chen et al. [38].

**Fig. 6:**
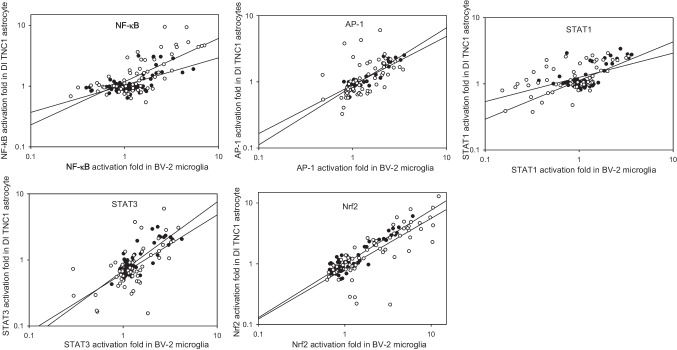
Correlations between TF responses to 64 flavonoids in BV-2 microglia (this paper) and DI TNC1 astrocytes (reference [29]). Closed and open circles correspond to 10 μM and 50 μM flavonoids, respectively. Each chart contains linear regression plots for both flavonoid concentrations

In order to demonstrate the applicability of the BV-2 microglia-based reporters for large-scale screening studies, we tested responses of the six transcription factors to a set of 64 flavonoids in BV-2 microglia. Since this set was used in our previous study to characterize neuroinflammation-related transcriptional responses to flavonoids in DI TNC1 astrocytes [29], it was possible to compare such responses between these two cell lines. As follows from Fig. 6, there is a consistent positive correlation between transcriptional responses to both 10 μM and 50 μM flavonoids in the microglia and astrocyte reporter lines. Indeed, tested representatives of three structural classes of flavonoids, namely flavanols (##1–6), flavonols (##36–49), and anthocyan(id)ins (##59–64), acted preferentially as inhibitors across multiple signaling pathways, both in BV2 microglia (Fig. 3) and DI TNC1 astrocytes [29]. In both types of cells, isoflavones (##50–58) and a subset of flavones (##14–26) consistently stimulated the transcriptional responses over the basal levels, with several polyphenols, such as flavone, 7-OMe-flavone (#16), 4′,7-(OMe)_2_-flavone (#19), chrysin, acacetin, daidzein, formononetin, genistein, and biochanin A, acting as the pan-inducers at both 10 μM and 50 μM concentrations. Notably, while in DI TNC1 astrocytes the STAT3 was the most sensitive TF to the inhibition by flavonoids as a whole group [29], in BV2 microglia it was the STAT1 whose activity was suppressed by the largest number of flavonoids at 50 μM (Fig. 3).

**Fig. 7:**
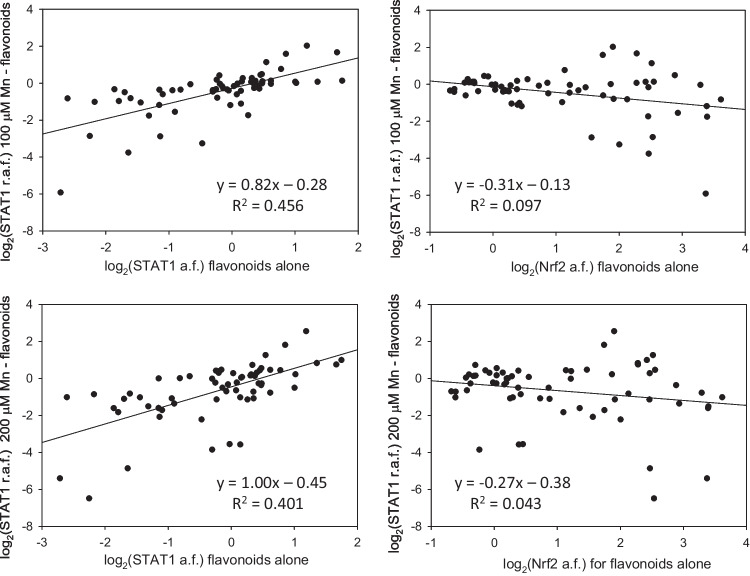
Correlations between the STAT1 responses to manganese/flavonoids combinations and the STAT1 or Nrf2 responses to flavonoids alone in BV-2 microglia. Sixty four flavonoids were presented to cells at 50 μM in all experiments, Mn(II) - at 100 μM (the upper row) or at 200 μM (the lower row). The activation folds (a.f.) are luciferase activities in flavonoid-only treated cells normalized for the untreated reporter; the relative activation folds (r.a.f.) are luciferase activities in Mn(II)/flavonoid-treated cells normalized for the Mn(II)-only treated reporter

In this study, we also demonstrated the utility of the microglia-based TF activation reporters in search for potential antidotes to neurotoxic and neuroinflammatory agents, exemplified in screening of manganese(II)—flavonoid combinations with the STAT1 reporters. Our data (Figure 4) suggest that, even though Mn(II) was presented to the cells in two- to fourfold excess over flavonoids, some polyphenols, such as ECG (#3), EGCG (#6), 3’,4’-(OH)_2_-flavone (#17), or myricetin (#48), could completely negate the cellular responses to the metal in BV2 microglia. There was a direct correlation between effects of flavonoids on the STAT1 activation in presence and in absence of Mn(II) (Fig. 7). No such correlation was found when the effects of flavonoids on the Nrf2 activation were compared with the effects of the combinations on the STAT1 activity. In addition, the interaction patterns for the Mn(II)/flavonoid and the IFNγ/flavonoid combinations were different (Fig. 4). These observations suggest inconsequential roles for metal chelation and antioxidant potential of flavonoids in their STAT1-affecting interactions with manganese (II) in BV-2 microglia.

To the best of our knowledge, this study and our previous report [29] are the first works attempting to assess an array of flavonoids in in vitro models of neuroinflammation. How our data compare to other studies on the neuroprotective potential of flavonoids? In one previous study [48], baicalin at 50 μM modestly decreased the STAT3 phosphorylation (1.2-fold) in amyloid-β treated BV-2 microglia. In our hands, 50 μM baicalin was a weak activator of the all six TFs (Fig. 3), but inhibited the 200 μM Mn(II)-induced STAT1 activation by twofold, along with improved viability of the Mn-treated cells (Fig. 4). Myricetin at 50 μM counteracted hypoxia-induced activation of BV-2 microglia, presumably by inhibiting the STAT1 phosphorylation [49]. In our experiments, 50 μM myricetin showed a weak, 1.2-fold, suppression of the basal STAT1 activation level, but a significant, 14-fold, inhibition of the 200 μM Mn(II)-induced STAT1 activation. Eriodictyol at 50 μM acted as an inducer of the Nrf2 in both BV-2 microglia and in brains of eriodictyol-fed mice [50]. As shown in Figure 3, this flavonoid (#9) at 50 μM activated the Nrf2 as well but acted as a weak inhibitor of the Nrf2 at 10 μM. These examples underscore an advantage of using stable reporters of transcriptional activation for rapid and effective generation of massive data on the cytoprotective potential of multiple flavonoids, as compared to the traditional one-compound-per-study approach. Furthermore, the later approach usually deals with one or limited number of flavonoid concentrations, which, for this class of reactive, multifunctional compounds, may create a problem of bias, due to a well-documented phenomenon of biphasic cellular responses to flavonoids [29, 51]. Therefore, profiling multiple flavonoids, or other sets of biologically interesting agents, by structure and dose, should provide for a systematic landscape of cell-signaling pathways for use in toxicology, pharmacology or nutrition areas. The insulated TF activation reporter transposon platform seems to be an appropriate tool up for the task.

## Conclusions

This study demonstrated the utility of insulated transcriptional activity reporter transposon for screening neurotoxic, neuroinflammatory, or neuroprotective agents in microglia. Screening of 12 metals in six reporters of the neuroinflammation-related signaling pathways exposed manganese (II) as a strong inducer of two transcription factors, homodimer STAT1 and heterodimer STAT1/STAT2 in microglia. Screening of an array of 64 flavonoids by this reporter set allowed mapping potentially pro- and anti-inflammatory effects of these agents at two fixed concentrations. Screening combinations of manganese(II) with the flavonoid array revealed potential inhibitors of the cytotoxic and pro-inflammatory activities of Mn(II) in microglia. Results of this in vitro study may inform future laboratory and clinical studies aiming at manganese neurotoxicology, as well as neuroprotective effects of dietary flavonoids.

## Supplementary Information

ESM 1 Cell viability and transcriptional factor induction folds; structures of flavonoids; dose-response curves (PDF). The online version contains supplementary material available at …

## Data Availability

Supporting data are included in the Supplementary Information. All other data are available from the corresponding author upon reasonable request.
