# Supplementary material for: Microglia Signaling Pathway Reporters Unveiled Manganese Activation of the Interferon/STAT1 Pathway and Its Mitigation by Flavonoids
Source: Mol Neurobiol. 2023 May 4;60(8):4679–92. doi: 10.1007/s12035-023-03369-w (PMC10293393; doi:10.1007/s12035-023-03369-w)
Supplement: Supplementary file 1 — Cell viability and transcriptional factor induction folds; structures of flavonoids; dose-response curves (PDF). The online version contains supplementary material available at … [file 12035_2023_3369_MOESM1_ESM.pdf]

Supporting Information to

Manganese elicited activation of the STAT1 in microglia with the interferon signaling pathway reporters can be mitigated by flavonoids.

Valeri V. Mossine[1,2]*, James K. Waters[2], Grace Y. Sun[1], Zezong Gu[3], and Thomas P. Mawhinney[1,2,4]

[1]*Department of Biochemistry, University of Missouri, Columbia, MO 65211, U.S.A.*

[2]*Agriculture Experiment Station Chemical Laboratories, University of Missouri, Columbia, MO 65211, U.S.A.*

[3]*Department of Pathology and Anatomical Sciences, University of Missouri, Columbia, MO 65211, U.S.A.*

[4]*Department of Child Health, University of Missouri, Columbia, MO 65211, U.S.A.*

*E-mail: mossinev@missouri.edu

Table S1. Structures of Flavonoids

Core	Code#	Trivial name	Substitutes	Source
Flavan-3-ols	1	(+)-catechin	3,3’,4’,5,7-$(OH)_5$	Sigma #C1251
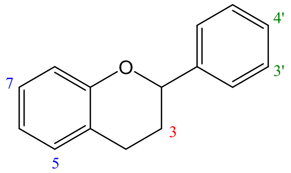	2	(-)-epicatechin	3,3’,4’,5,7-$(OH)_5$	Carl Roth KG #7293
	3	ECG	3-gallate,3’,4’,5,7-$(OH)_4$	Santa Cruz Bio #sc204739
	4	(-)-gallocatechin	3,3’,4’,5’,5,7-$(OH)_6$	Sigma #G6657
	5	(-)-epigallocatechin	3,3’,4’,5’,5,7-$(OH)_6$	Sigma #E3768
	6	EGCG	3-gallate,3’,4’,5’,5,7-$(OH)_5$	Enzo LifeSci #270263
Flavanones	7	(±)-naringenin	4’,5,7-$(OH)_3$	Sigma #N5893
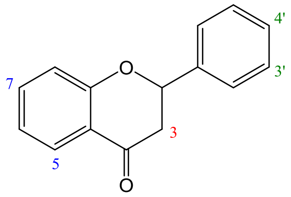	8	naringin	4’,5-$(OH)_2$,7-*O*-Rha(1,2)Glc	Sigma #N1376
	9	eriodictyol	3’,4’,5,7-$(OH)_4$	Sigma #94258
	10	hesperetin	3’,5,7-$(OH)_3$, 4’-OMe	TCI #H0721
	11	hesperidin	3’,5-$(OH)_2$, 4’-OMe, 7-*O*-Rha(1,6)Glc	AlfaAesar #J62126
	12	taxifolin	3,3’,4’,5,7-$(OH)_5$	Sigma #T4512
	13	silybin=silibinin	3,5,7-$(OH)_3$, 3’,4’-*O*,*O*-coniferyl	Chem Cruz #sc202812
Flavones	14	flavone		AlfaAesar #A13627
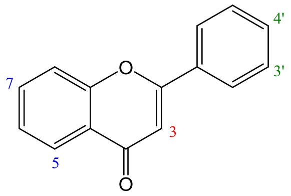	15	α-naphthoflavone	7,8-benzo	TCI #B0056
	16		7-OMe	AlfaAesar #B24850
	17		3’,4’-$(OH)_2$	AlfaAesar #L14161
	18		4’,5-$(OH)_2$	AlfaAesar #H27650
	19		4’,7-$(OMe)_2$	Indofine #D431
	20		5,6-$(OH)_2$	AlfaAesar #H27039
	21	chrysin	5,7-$(OH)_2$	Acros #11032
	22		7,8-$(OH)_2$	Sigma #D5446
	23		2’,3’,6-$(OH)_3$	Indofine #22-324
	24		3’,7,8-$(OH)_3$	Indofine #T411
	25	apigenin	4’,5,7-$(OH)_3$	Indofine #A002
	26	acacetin	4’-OMe, 5,7-$(OH)_2$	TCI #A3013
	27		4’,7,8-$(OH)_3$	Indofine #T412
	28	baicalein	5,6,7-$(OH)_3$	Aldrich #455119
	29	baicalin	5,6-$(OH)_2$, 7-*O*-Glc	Indofine #06-012
	30	wogonin	5,7-$(OH)_2$, 8-OMe	Sigma #W0769
	31	luteolin	3’,4’,5,7-$(OH)_4$	TSZ Chem #RRL02
	32	diosmin	3’,5-$(OH)_2$, 4’-OMe, 7-*O*-Rha(1,6)Glc	AlfaAesar #J62073
	33	neodiosmin	3’,5-$(OH)_2$, 4’-OMe, 7-*O*-Rha(1,2)Glc	Indofine #021249
	34	vitexin	4’,5,7-$(OH)_3$, 8-*C*-Glc	Adipogen #AGCN20425
	35	tangeretin	4’,5,6,7,8-$(OMe)_5$	Indofine #06-026
	36	flavonol	3-OH	Eastman #6585
	37		3-OH, 4’-OMe	TCI #H1405
	38	galangin	3,5,7-$(OH)_3$	AlfaAesar #L14223
	39	fisetin	3,3’,4’,7-$(OH)_4$	Indofine #F002
	40	kaempferol	3,4’,5,7-$(OH)_4$	AlfaAesar #J60373
	41		3,4’,7,8-$(OH)_4$	AlfaAesar #H27348
	42	morin	3,2’,4’,5,7-$(OH)_5$	Acros #35400
	43	quercetin	3,3’,4’,5,7-$(OH)_5$	Sigma #Q0125

	44	rhamnetin	3,3’,4’,5-(OH)$_4$,7-OMe	Santa Cruz Bio #sc236668
	45	quercetin-3-glucoside	3-*O*-βGlc, 3’,4’,5,7-(OH)$_4$	Sigma #17793
	46	quercitrin	3-*O*-αRha, 3’,4’,5,7-(OH)$_4$	Sigma #Q3001
	47	rutin	3-*O*-Rha(1,6)Glc 3’,4’,5,7-(OH)$_4$	Sigma #R9000
	48	myricetin	3,3’,4’,5’,5,7-(OH)$_6$	Sigma #M6760
	49	myricitrin	3-*O*-αRha, 3’,4’,5’,5,7-(OH)$_5$	Aldrich #CDS006718
Isoflavones	50	daidzein	4’,7-(OH)$_2$	Sigma #D7802
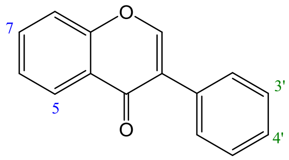	51	daidzin	4’,7-*O*-βGlc	TSZ Chem #RD02
	52	formononetin	4’-OMe, 7-OH	Aldrich #94334
	53		3’,4’,7-(OH)$_3$	Indofine #T415
	54	genistein	4’,5,7-(OH)$_3$	Sigma #G6649
	55	genistin	4’,5-(OH)$_2$, 7-*O*-βGlc	TSZ Chem #RG01
	56	prunetin	4’,5-(OH)$_2$, 7-OMe	Sigma #82415
	57	biochanin A	4’-OMe, 5,7-(OH)$_2$	Sigma #D2016
	58	puerarin	4’,7-(OH)$_2$, 8-*C*-Glc	AlfaAesar #J66528
Anthocyan(id)ins	59	pelargonin	3,5-(*O*-βGlc)$_2$, 4’,7-(OH)$_2$	Sigma #52926
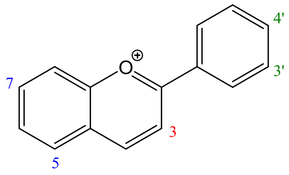	60	cyanidin	3,3’,4’,5,7-(OH)$_5$	Sigma #79457
	61	keracyanin	3-*O*-Rha(1,6)Glc, 3’,4’,5,7-(OH)$_4$	Sigma #36428
	62	cyanin	3,5-(*O*-βGlc)$_2$, 3’,4’,7-(OH)$_3$	Sigma #94258
	63	delphinidin	3,3’,4’,5’,5,7-(OH)$_6$	Sigma #74397
	64	oenin	3-*O*-βGlc, 3’,5’-(OMe)$_2$, 4’,5,7-(OH)$_3$	Carl Roth KG #4006

Table S2. Viability and transcriptional factor induction in BV-2 microglia-derived reporter cells treated with pathway activators/ inhibitors for 18 hours. SD values* for the last 2 digits are given in parentheses.

Inducer agent	Relative **viability**	Relative **NF-κB** fold	Relative **AP-1** fold	Relative **STAT1** fold	Relative **STAT1/2** fold	Relative **STAT3** fold	Relative **Nrf2** fold	Relative **MTF-1** fold
5 ng/mL **TNFα**	0.943 (51)	7.29 (20)	0.903 (56)	2.24 (13)	1.83 (29)	0.485 (85)	1.62 (26)	0.457 (25)
100 ng/mL **LPS**	1.01 (10)	29.8 (27)	0.635 (82)	2.06 (29)	2.86 (39)	0.364 (57)	5.59 (71)	0.170 (38)
5 µg/mL **poly(I:C)**	0.852 (92)	4.40 (39)	0.84 (14)	0.828 (88)	2.48 (61)	0.746 (90)	1.26 (17)	0.559 (39)
100 ng/mL **PMA**	1.128 (66)	1.27 (29)	1.95 (13)	0.74 (14)	1.28 (38)	0.600 (36)	3.81 (50)	0.80 (12)
500 nM **CDDO-Me**	0.83 (11)	0.061 (06)	0.231 (09)	0.139 (23)	2.18 (40)	0.743 (35)	27.2 (20)	0.323 (17)
10 µM **tBHQ**	0.88 (12)	0.492 (35)	0.907 (57)	0.467 (34)	5.52 (94)	1.07 (21)	25.9 (15)	0.839 (91)
250 µM **H_2O_2**	1.020 (38)	0.818 (68)	0.560 (43)	0.675 (71)	1.96 (31)	0.892 (36)	4.52 (33)	0.273 (10)
60 µM **Pyocyanin**	0.91 (10)	1.039 (77)	0.97 (16)	1.04 (10)	2.88 (56)	2.74 (86)	31.5 (21)	0.993 (69)
20 µM ***o*-phenanthroline**	0.88 (12)	0.604 (45)	0.260 (22)	0.327 (17)	0.333 (43)	1.65 (18)	9.98 (67)	0.341 (22)
20 µM **$ZnCl_2$**	1.05 (17)	1.08 (12)	0.990 (90)	0.808 (45)	0.958 (80)	0.879 (84)	3.94 (34)	5.35 (41)
10 ng/mL **IFN-α**	0.991 (32)	0.80 (11)	1.01 (15)	6.26 (42)	6.65 (83)	2.11 (20)	0.773 (77)	1.033 (25)
5 ng/mL **IFN-γ**	1.08 (10)	1.35 (29)	0.465 (83)	193. (14)	10.6 (19)	14.02 (70)	0.162 (34)	0.093 (02)
10 ng/mL **IL-6**	0.981 (51)	0.99 (10)	0.872 (53)	1.09 (11)	0.91 (13)	9.30 (12)	0.784 (65)	1.01 (10)
25 ng/mL **IL-4**	0.993 (44)	0.65 (11)	1.02 (12)	0.498 (68)	0.740 (83)	1.26 (19)	0.828 (21)	1.054 (95)
200 nM **Dasatinib**	0.75 (13)	0.436 (87)	0.771 (80)	1.06 (13)	1.03 (23)	0.611 (40)	0.466 (40)	0.580 (55)
200 nM **Ruxolitinib**	1.040 (74)	0.68 (13)	0.986 (76)	0.097 (07)	0.958 (71)	0.430 (29)	0.818 (56)	0.989 (73)
50 µM **AG490**	1.10 (13)	0.052 (04)	0.575 (65)	0.059 (05)	1.68 (19)	0.443 (83)	16.9 (24)	0.839 (62)

*For viability, n=21; for specific TFs, n=3.

Table S3. TF induction fold and viability (GFP expression) in BV-2 microglia-based reporters for transcriptional activation of homodimer STAT1 and heterodimer STAT1/STAT2. The cells were treated with Mn(II) for 12 hours, n=3.

	STAT1				STAT1/STAT2			
Mn^{2+}, μM	Fold	SD	GFP	SD	Fold	SD	GFP	SD
0	1.05	0.12	0.98	0.03	0.98	0.02	0.99	0.06
21	0.98	0.13	0.97	0.01	1.11	0.16	0.95	0.07
30	0.98	0.15	0.95	0.06	1.14	0.12	0.95	0.03
42	1.02	0.10	0.95	0.03	1.39	0.32	0.94	0.03
58	1.10	0.11	1.02	0.05	1.65	0.36	0.98	0.02
82	4.03	1.18	1.01	0.06	3.49	0.94	0.97	0.03
114	16.13	1.89	1.02	0.04	17.5	2.0	0.99	0.02
160	17.71	2.21	0.90	0.08	23.1	2.5	0.92	0.06

Table S4. Relative TF induction fold in BV-2 microglia-based reporters for transcriptional activation of STAT1 and STAT1/STAT2. The cells were treated with combinations of Mn(II) and IFNα for 12 hours, the data points are single measurements. For each row, the relative activation fold is calculated as TF activation fold divided by the respective TF activation fold in Mn(II)-only treated cells (Table S2). Values highlighted in yellow indicate weak synergistic interactions between Mn(II) and IFNα.

IFNα, ng/mL	0	0.2	0.34	0.56	0.93	1.56	2.6	4.3	7.2	12	20
Mn^{2+}, μM	Relative STAT1 activation fold										
0	1.00	1.19	1.14	1.28	1.24	1.33	1.65	2.01	2.50	3.03	4.07
21	1.00	1.21	0.92	1.07	1.12	1.56	1.51	1.97	2.55	3.20	4.10
30	1.00	1.11	0.92	1.21	1.20	1.23	1.62	1.97	2.62	3.28	4.26
42	1.00	0.93	1.10	1.23	1.09	1.25	1.50	2.01	2.83	3.89	4.16
58	1.00	1.24	1.24	1.05	0.93	1.53	1.43	1.66	2.71	3.26	3.46
82	1.00	1.03	0.99	1.37	1.47	1.56	1.60	1.66	2.03	3.51	3.14
114	1.00	1.10	0.83	0.98	1.34	1.10	0.94	1.14	1.07	1.13	1.65
160	1.00	0.90	0.91	1.05	0.95	1.23	1.04	1.30	1.23	1.45	1.09
	Relative STAT1/STAT2 activation fold										
0	1.00	1.67	2.45	2.42	2.74	3.49	3.86	4.31	5.33	6.07	6.29
21	1.00	1.32	1.69	1.75	2.39	2.52	2.65	3.56	4.06	4.60	5.03
30	1.00	1.29	1.53	1.85	2.20	2.31	2.74	3.03	3.89	3.89	4.85
42	1.00	1.17	1.11	1.72	1.80	1.83	2.37	2.48	2.92	3.30	3.25
58	1.00	2.36	1.90	2.25	2.33	3.04	3.30	3.20	4.82	4.80	4.47
82	1.00	1.56	1.97	1.58	1.54	1.36	1.33	1.75	1.56	1.94	1.77
114	1.00	1.07	1.24	0.91	1.01	0.90	0.93	1.18	1.14	1.01	0.91
160	1.00	1.19	1.14	1.31	1.27	1.00	1.03	1.08	1.00	1.10	1.01

Table S5. Relative TF induction fold in BV-2 microglia-based reporters treated with combinations of Mn(II) and IFNγ for 12 hours, the data points are single measurements. Values highlighted in yellow indicate weak synergistic interactions between Mn(II) and IFNγ.

IFNγ, ng/mL	0	0.004	0.008	0.016	0.032	0.063	0.125	0.25	0.5	1	2
Mn^{2+}, μM	STAT1 activation fold										
0	1.00	1.18	1.30	2.45	4.16	9.03	18.0	29.4	44.9	53.7	60.3
21	1.00	1.08	1.23	1.98	4.20	8.75	14.7	27.7	39.2	49.1	55.1
30	1.00	1.01	1.01	2.17	4.15	7.62	13.0	24.6	36.1	44.5	55.1
42	1.00	1.32	1.28	1.92	3.45	8.01	13.5	23.8	38.9	38.6	49.9
58	1.00	1.12	1.87	2.32	3.40	9.35	11.4	23.1	36.5	39.9	46.8
82	1.00	1.46	1.62	3.34	4.12	5.68	7.52	10.8	16.2	18.2	24.7
114	1.00	1.32	1.58	1.68	1.79	1.69	2.02	2.55	2.82	2.93	3.54
160	1.00	1.08	1.10	1.12	1.12	1.28	1.63	2.01	1.92	2.20	1.86
	STAT1/STAT2 activation fold										
0	1.00	1.28	1.59	2.70	4.16	7.59	13.46	16.69	22.25	23.65	22.42
21	1.00	1.18	1.68	2.60	4.46	8.30	11.95	17.54	22.63	23.03	21.46
30	1.00	1.38	1.65	2.91	4.77	6.94	12.46	17.29	21.78	20.20	21.34
42	1.00	1.50	1.41	2.64	5.26	7.49	11.96	17.71	21.50	21.11	19.97
58	1.00	1.20	1.65	2.13	3.17	5.03	7.76	10.56	15.65	12.33	11.48
82	1.00	1.28	1.26	2.11	2.72	3.66	5.30	4.77	5.43	5.24	4.72
114	1.00	0.98	1.22	1.32	1.65	1.57	1.76	1.87	1.64	1.27	1.16
160	1.00	1.04	0.95	1.59	1.73	1.73	1.87	1.94	1.71	1.57	1.15

Table S6. Relative TF induction fold in BV-2 microglia-based reporters treated with combinations of Mn(II) and A$\beta_{25\text{-}35}$ for 12 hours, the data points are single measurements. Values highlighted in yellow or blue indicate, respectively, weak potentiation or inhibition of Mn(II) by A$\beta_{25\text{-}35}$.

A$\beta_{25\text{-}35}$, µM	0	0.098	0.195	0.39	0.78	1.56	3.13	6.25	12.5	25	50
Mn^{2+}, µM	STAT1 activation fold										
0	1.00	0.98	0.96	1.00	1.06	1.21	1.09	0.81	0.98	0.95	0.99
21	1.00	0.92	0.97	1.10	1.11	1.24	1.15	1.43	1.26	1.10	0.98
30	1.00	0.86	1.18	0.70	0.83	0.89	0.95	1.11	1.03	1.11	1.04
42	1.00	1.04	0.82	1.30	1.07	0.89	1.00	0.86	1.09	1.09	1.16
58	1.00	1.25	1.03	1.11	1.16	1.06	0.96	0.95	1.09	1.11	1.14
82	1.00	1.13	1.23	1.34	1.37	1.42	1.10	1.00	0.51	0.56	0.50
114	1.00	0.93	1.11	1.04	1.30	1.12	1.28	1.29	1.14	0.79	0.79
160	1.00	0.93	0.72	0.91	0.88	0.99	0.95	1.08	1.18	1.04	0.91
	STAT1/STAT2 activation fold										
0	1.00	1.00	1.14	1.09	1.22	1.00	1.18	1.18	1.16	1.08	1.18
21	1.00	0.89	0.99	0.99	0.95	0.95	1.15	1.00	1.24	0.92	0.92
30	1.00	0.90	0.91	1.10	1.02	0.98	0.95	0.89	0.84	1.00	0.92
42	1.00	0.91	0.75	0.96	1.04	1.14	0.89	0.94	1.31	1.10	1.80
58	1.00	0.78	1.19	1.01	1.22	1.18	0.83	1.23	1.24	0.82	1.61
82	1.00	0.85	0.91	1.34	0.89	1.04	1.10	0.81	1.48	1.03	1.43
114	1.00	0.78	1.42	0.81	1.03	1.02	1.12	0.92	0.93	0.80	1.13
160	1.00	0.93	0.97	1.00	1.24	1.15	1.14	0.94	0.80	0.64	0.84

Table S7. Relative TF induction fold in BV-2 microglia-based reporters treated with combinations of Mn(II) and poly(I:C) for 12 hours, the data points are single measurements.

Poly(I:C), µg/mL	0	2	8	32	128	512		0	2	8	32	128	512
Mn^{2+}, µM	STAT1 activation fold							STAT1/STAT2 activation fold					
0	1.00	13.2	15.9	17.1	17.3	18.2		1.00	9.45	13.5	17.1	17.9	18.5
21	1.00	9.33	13.3	11.4	12.6	13.5		1.00	6.56	9.05	9.37	10.7	11.9
30	1.00	10.6	11.7	12.0	14.3	15.6		1.00	6.90	8.93	10.6	11.4	13.7
42	1.00	7.99	9.70	9.59	11.7	11.4		1.00	4.55	5.03	8.51	9.04	10.9
58	1.00	8.90	10.0	11.6	12.2	14.6		1.00	3.77	4.94	7.19	6.85	8.90
82	1.00	2.58	2.24	1.95	2.10	2.33		1.00	3.51	4.20	5.34	5.85	6.38
114	1.00	1.20	1.18	1.12	1.03	1.19		1.00	1.41	1.48	1.76	1.51	1.64
160	1.00	1.06	1.08	1.00	1.08	1.07		1.00	0.99	1.10	1.01	1.03	0.93

Table S8. Viability and transcriptional factor induction in BV-2 microglia-derived reporter cells treated with flavonoids for 18 hours. For viability, n=18; for specific TFs, n=3.

			GFP fluorescence		NF-κB		AP-1		STAT1		STAT1/2		STAT3		Nrf2	
Code #	name	conc	Fold	SD	Fold	SD	Fold	SD	Fold	SD	Fold	SD	Fold	SD	Fold	SD
1	catechin	10 μM	0.963	0.091	1.928	0.339	0.901	0.061	1.164	0.178	1.150	0.222	0.950	0.137	0.716	0.030
		50 μM	1.050	0.069	1.476	0.127	0.894	0.103	1.276	0.188	0.951	0.108	1.199	0.023	0.655	0.057
2	epicatechin	10 μM	1.150	0.083	1.567	0.362	0.870	0.057	1.044	0.107	0.865	0.185	1.103	0.156	0.668	0.116
		50 μM	1.113	0.084	1.214	0.370	0.865	0.055	1.104	0.112	0.960	0.120	1.392	0.036	0.653	0.028
3	ECG	10 μM	1.145	0.050	1.440	0.339	0.864	0.040	0.943	0.146	0.849	0.123	1.083	0.083	0.950	0.084
		50 μM	1.197	0.124	1.384	0.188	0.773	0.034	0.982	0.067	1.176	0.147	1.004	0.067	1.371	0.035
4	GC	10 μM	1.154	0.085	1.247	0.297	0.939	0.046	1.086	0.173	0.898	0.147	1.116	0.187	0.837	0.063
		50 μM	1.133	0.069	1.041	0.134	0.883	0.050	0.909	0.185	0.818	0.161	1.123	0.148	1.301	0.107
5	EGC	10 μM	1.131	0.085	0.975	0.140	0.943	0.085	1.074	0.126	0.899	0.165	1.135	0.141	0.670	0.037
		50 μM	1.222	0.055	1.217	0.242	0.851	0.057	0.948	0.204	0.757	0.116	1.225	0.068	0.624	0.012
6	EGCG	10 μM	1.113	0.087	1.116	0.140	0.913	0.016	1.040	0.078	0.916	0.104	1.012	0.103	0.897	0.086
		50 μM	1.156	0.078	0.943	0.206	0.799	0.021	1.104	0.228	0.864	0.134	1.000	0.153	1.311	0.196
7	naringenin	10 μM	1.084	0.055	1.306	0.352	1.121	0.124	1.188	0.220	1.055	0.044	1.054	0.125	1.145	0.247
		50 μM	1.013	0.076	1.517	0.283	1.723	0.095	1.715	0.261	1.710	0.271	2.408	0.079	2.199	0.070
8	naringin	10 μM	0.937	0.115	1.388	0.142	1.077	0.041	0.894	0.131	0.739	0.105	0.922	0.153	1.090	0.104
		50 μM	1.063	0.084	1.318	0.105	0.955	0.030	0.872	0.162	1.106	0.187	0.985	0.038	0.948	0.042
9	eriodictyol	10 μM	1.048	0.059	1.068	0.105	0.994	0.038	0.532	0.028	0.678	0.147	1.323	0.048	0.809	0.020
		50 μM	0.899	0.069	1.677	0.219	1.266	0.034	0.289	0.115	1.199	0.251	0.926	0.040	2.138	0.118
10	hesperetin	10 μM	1.127	0.052	1.046	0.141	1.084	0.010	0.954	0.189	0.969	0.159	1.534	0.080	1.495	0.083
		50 μM	1.068	0.067	3.176	0.406	2.733	0.063	1.810	0.193	2.484	0.425	3.227	0.126	3.344	0.240
11	hesperidin	10 μM	1.192	0.022	1.167	0.046	1.029	0.035	0.718	0.113	0.796	0.045	1.112	0.041	0.830	0.030
		50 μM	0.997	0.057	1.225	0.157	1.108	0.046	1.351	0.239	1.149	0.107	1.172	0.076	1.024	0.060
12	taxifolin	10 μM	1.182	0.035	1.251	0.165	1.006	0.011	1.019	0.103	0.771	0.061	1.216	0.100	0.692	0.028
		50 μM	1.046	0.067	1.028	0.107	1.124	0.052	1.055	0.162	1.139	0.084	1.399	0.190	0.740	0.029
13	silybin=silibinin	10 μM	1.141	0.043	0.944	0.103	1.028	0.039	1.127	0.129	0.869	0.144	1.135	0.136	0.962	0.098
		50 μM	1.040	0.036	0.868	0.182	1.165	0.028	0.846	0.064	0.944	0.055	0.943	0.071	1.304	0.081
14	flavone	10 μM	1.064	0.047	4.122	0.686	2.422	0.333	2.755	0.573	2.435	0.541	3.194	0.201	2.998	0.328
		50 μM	0.902	0.072	6.091	1.068	2.061	0.148	1.458	0.228	1.774	0.438	3.864	0.321	5.723	0.878
15	α-naphthoflavone	10 μM	0.976	0.144	5.379	0.417	1.762	0.192	1.853	0.208	1.204	0.204	1.412	0.136	2.211	0.046
		50 μM	0.299	0.071	3.529	0.614	0.478	0.056	0.210	0.161	0.665	0.171	0.297	0.160	5.788	0.305

16	7-OMe-flavone	10 µM	0.956	0.077	3.272	0.691	2.487	0.093	3.374	0.713	2.574	0.602	2.331	0.154	2.503	0.142
		50 µM	0.796	0.097	7.060	1.633	2.147	0.045	2.560	0.794	2.349	0.297	2.718	0.261	4.845	0.249
17	3’,4’-(OH)2-flavone	10 µM	1.081	0.108	1.132	0.141	1.412	0.068	0.373	0.103	1.082	0.118	1.215	0.084	1.439	0.066
		50 µM	0.838	0.092	1.067	0.205	0.815	0.042	0.320	0.075	0.640	0.145	0.531	0.068	5.537	0.145
18	4’,5-(OH)2-flavone	10 µM	1.055	0.131	2.027	0.177	2.180	0.216	1.377	0.225	1.568	0.030	2.141	0.067	3.775	0.111
		50 µM	0.862	0.115	2.666	0.642	1.430	0.036	0.534	0.063	1.131	0.116	0.972	0.110	7.635	0.694
19	4’,7-(OMe)2-flavone	10 µM	1.058	0.113	2.416	0.302	2.521	0.014	1.874	0.011	2.299	0.454	2.425	0.276	3.022	0.364
		50 µM	0.646	0.162	6.612	1.008	2.189	0.422	1.022	0.113	1.403	0.087	1.336	0.205	5.522	0.736
20	5,6-(OH)2-flavone	10 µM	1.093	0.142	1.588	0.321	1.975	0.132	1.176	0.206	1.645	0.111	2.075	0.202	1.923	0.071
		50 µM	0.757	0.115	1.373	0.221	1.228	0.056	0.164	0.053	0.880	0.153	0.523	0.049	12.245	1.638
21	chrysin	10 µM	1.073	0.134	1.894	0.215	2.419	0.004	2.531	0.213	3.015	0.586	2.711	0.160	2.828	0.196
		50 µM	1.007	0.136	3.167	0.702	2.526	0.319	2.026	0.087	2.993	0.110	2.375	0.125	3.649	0.383
22	7,8-(OH)2-flavone	10 µM	1.103	0.126	1.066	0.204	1.214	0.058	0.921	0.122	1.027	0.190	1.371	0.104	0.853	0.054
		50 µM	0.945	0.118	1.128	0.282	1.524	0.018	0.454	0.063	1.562	0.184	1.518	0.065	2.955	0.161
23	2',3',6-(OH)3-flavone	10 µM	1.006	0.121	1.655	0.449	1.026	0.060	1.030	0.142	1.148	0.110	1.033	0.171	0.725	0.052
		50 µM	0.998	0.143	1.447	0.136	1.957	0.134	0.851	0.159	1.588	0.302	1.830	0.014	3.767	0.355
24	3',7,8-(OH)3-flavone	10 µM	1.074	0.077	0.898	0.151	0.995	0.030	0.860	0.153	0.868	0.147	1.103	0.067	0.674	0.029
		50 µM	0.969	0.072	0.993	0.109	1.012	0.037	0.367	0.027	0.758	0.217	0.985	0.060	1.226	0.139
25	apigenin	10 µM	1.093	0.081	1.980	0.202	2.416	0.026	1.311	0.270	2.548	0.393	2.043	0.148	4.227	0.934
		50 µM	0.806	0.108	4.558	0.922	2.095	0.181	0.722	0.016	1.783	0.528	1.051	0.064	4.011	0.146
26	acacetin	10 µM	1.151	0.115	2.494	0.100	3.231	0.232	2.165	0.219	2.715	0.293	2.826	0.365	3.836	0.246
		50 µM	0.842	0.116	4.751	0.444	3.438	0.406	1.196	0.138	3.031	0.290	1.973	0.047	5.508	0.293
27	4',7,8-(OH)3-flavone	10 µM	1.168	0.115	0.808	0.133	0.986	0.022	0.944	0.024	0.862	0.182	1.157	0.151	0.686	0.070
		50 µM	1.000	0.108	0.882	0.116	1.017	0.067	0.965	0.049	0.979	0.171	1.230	0.175	1.118	0.096
28	baicalein	10 µM	1.120	0.136	0.769	0.194	1.316	0.094	1.096	0.243	1.308	0.199	1.281	0.130	0.691	0.090
		50 µM	1.014	0.127	1.146	0.170	1.788	0.095	0.811	0.164	1.337	0.332	1.600	0.147	2.319	0.146
29	baicalin	10 µM	1.078	0.098	1.001	0.202	1.498	0.010	1.110	0.232	0.958	0.221	1.073	0.026	0.703	0.011
		50 µM	1.045	0.124	1.428	0.251	2.182	0.054	1.278	0.134	1.776	0.092	1.521	0.233	1.656	0.103
30	wogonin	10 µM	1.077	0.083	0.815	0.132	0.903	0.057	0.677	0.212	0.819	0.023	1.010	0.075	1.058	0.123
		50 µM	0.785	0.111	1.604	0.213	1.470	0.081	0.876	0.119	1.617	0.242	1.639	0.108	2.327	0.379
31	luteolin	10 µM	0.883	0.154	1.145	0.104	1.176	0.202	0.769	0.144	0.543	0.049	0.755	0.164	1.114	0.145
		50 µM	0.734	0.146	2.071	0.108	1.737	0.054	0.328	0.052	1.047	0.174	0.508	0.099	4.372	0.128
32	diosmin	10 µM	1.052	0.136	0.964	0.165	0.941	0.042	1.061	0.073	0.395	0.138	0.953	0.166	0.911	0.134
		50 µM	1.187	0.151	0.585	0.197	0.912	0.053	0.840	0.052	1.089	0.202	0.825	0.172	1.139	0.054
33	neodiosmin	10 µM	1.057	0.093	1.071	0.193	1.002	0.037	1.254	0.101	0.669	0.130	0.935	0.191	0.849	0.126

		50 µM	1.067	0.064	0.817	0.141	1.005	0.059	1.235	0.201	0.920	0.136	1.247	0.155	0.814	0.024
34	vitexin	10 µM	1.117	0.080	0.888	0.136	0.951	0.060	1.429	0.340	0.722	0.113	1.055	0.088	0.826	0.031
		50 µM	1.094	0.091	1.216	0.487	0.988	0.103	1.262	0.342	0.853	0.067	1.257	0.202	0.812	0.035
35	tangeretin	10 µM	1.073	0.073	0.701	0.309	1.214	0.111	1.600	0.236	1.101	0.371	1.257	0.160	1.433	0.201
		50 µM	0.954	0.054	1.749	0.358	1.440	0.067	0.898	0.046	0.944	0.473	1.587	0.147	2.767	0.362
36	flavonol	10 µM	1.051	0.075	0.794	0.110	0.999	0.047	1.127	0.140	0.769	0.130	1.298	0.085	1.270	0.182
		50 µM	0.594	0.079	2.663	0.960	1.894	0.190	0.152	0.074	1.543	0.441	1.283	0.129	10.364	1.260
37	4’-OMe-flavonol	10 µM	1.103	0.057	1.307	0.545	1.019	0.045	1.202	0.100	0.663	0.140	1.022	0.017	1.299	0.609
		50 µM	1.005	0.097	0.987	0.354	1.187	0.019	2.005	0.185	1.153	0.219	1.295	0.094	1.818	0.175
38	galangin	10 µM	0.981	0.088	0.990	0.131	0.874	0.087	1.161	0.290	0.826	0.109	1.028	0.044	1.882	0.230
		50 µM	0.973	0.118	2.095	0.345	2.374	0.172	1.389	0.259	1.722	0.182	2.421	0.112	7.381	0.068
39	fisetin	10 µM	1.077	0.027	0.839	0.344	1.059	0.040	0.798	0.018	0.794	0.200	1.235	0.044	0.998	0.079
		50 µM	0.932	0.098	1.094	0.169	1.534	0.147	0.275	0.061	1.739	0.222	0.960	0.304	2.557	0.035
40	kaempferol	10 µM	1.107	0.083	1.007	0.596	1.030	0.028	0.756	0.040	0.794	0.071	1.144	0.153	1.794	0.044
		50 µM	0.963	0.115	2.292	0.395	2.156	0.077	1.535	0.112	1.889	0.414	3.077	0.708	9.778	0.183
41	4',7,8-(OH)3-flavonol	10 µM	1.142	0.080	0.820	0.146	0.957	0.026	1.223	0.084	0.617	0.146	1.191	0.079	0.793	0.041
		50 µM	1.122	0.167	0.642	0.176	0.886	0.055	0.221	0.075	1.911	0.154	0.740	0.030	1.338	0.026
42	morin	10 µM	1.150	0.059	0.723	0.173	0.994	0.057	1.027	0.070	0.588	0.130	1.214	0.038	0.823	0.022
		50 µM	1.028	0.061	0.892	0.095	1.174	0.101	1.326	0.184	0.862	0.237	1.427	0.125	1.098	0.061
43	quercetin	10 µM	1.061	0.059	0.639	0.099	1.016	0.046	1.302	0.154	0.908	0.058	1.518	0.196	0.832	0.072
		50 µM	0.959	0.103	0.743	0.270	1.220	0.010	0.520	0.062	1.066	0.125	1.471	0.027	1.191	0.028
44	rhamnetin	10 µM	1.146	0.146	0.447	0.147	0.849	0.028	1.149	0.355	0.642	0.156	1.230	0.020	0.922	0.058
		50 µM	1.253	0.300	0.319	0.038	0.483	0.150	0.308	0.124	0.608	0.146	1.227	0.100	1.836	0.093
45	quercetin-3-glucoside	10 µM	1.069	0.092	0.942	0.093	0.883	0.062	0.881	0.187	0.689	0.132	1.102	0.095	0.787	0.054
		50 µM	0.710	0.110	1.753	0.337	0.922	0.047	0.467	0.187	0.790	0.185	0.293	0.118	3.357	0.068
46	quercitrin	10 µM	0.916	0.042	1.543	0.909	0.879	0.162	1.165	0.345	1.771	0.508	0.989	0.097	0.888	0.088
		50 µM	1.002	0.063	0.606	0.309	1.026	0.073	1.063	0.152	1.992	0.791	1.254	0.107	0.987	0.010
47	rutin	10 µM	1.090	0.094	1.125	0.625	0.901	0.150	0.840	0.176	1.486	0.183	1.025	0.176	0.846	0.122
		50 µM	1.078	0.129	0.676	0.313	0.930	0.061	1.397	0.226	1.933	0.156	1.245	0.064	0.770	0.055
48	myricetin	10 µM	1.048	0.072	0.684	0.541	0.866	0.104	0.903	0.116	1.650	0.061	1.060	0.130	0.849	0.092
		50 µM	1.130	0.099	0.266	0.222	0.757	0.032	0.811	0.618	1.214	0.210	1.036	0.055	0.850	0.049
49	myricitrin	10 µM	1.092	0.093	0.816	0.149	0.965	0.092	0.904	0.303	1.692	0.297	1.138	0.170	0.790	0.021
		50 µM	1.117	0.107	0.516	0.201	0.851	0.143	1.115	0.447	1.323	0.611	1.262	0.058	0.733	0.050
50	daidzein	10 µM	1.064	0.076	1.575	0.658	2.393	0.206	2.253	0.720	3.172	0.459	2.481	0.107	3.233	0.324
		50 µM	1.117	0.130	1.649	0.248	2.237	0.346	3.172	0.650	2.889	0.170	2.867	0.246	4.855	0.123

51	daidzin	10 µM	1.055	0.087	1.391	1.082	1.012	0.152	0.766	0.356	1.443	0.093	1.046	0.103	0.876	0.054
		50 µM	1.068	0.071	0.437	0.131	0.948	0.109	1.368	0.755	1.775	0.703	1.300	0.080	0.907	0.104
52	formononetin	10 µM	1.062	0.081	2.156	0.607	3.526	0.482	3.627	0.769	3.812	1.459	4.207	0.316	4.014	0.672
		50 µM	0.998	0.077	2.603	0.614	2.839	0.045	2.277	1.197	4.583	2.426	3.430	0.395	3.735	0.082
53	3',4',7-(OH)3-isoflavone	10 µM	0.842	0.132	0.887	0.903	1.390	0.038	0.619	0.484	0.920	0.544	1.078	0.042	1.698	0.180
		50 µM	0.903	0.107	1.344	0.159	1.427	0.133	0.449	0.342	2.668	0.649	1.262	0.013	10.482	1.323
54	genistein	10 µM	1.098	0.103	2.169	0.470	2.180	0.216	1.943	0.410	2.911	0.417	2.325	0.071	3.624	0.289
		50 µM	0.850	0.083	2.445	1.034	2.098	0.267	1.536	0.776	3.235	0.478	1.563	0.104	5.859	0.099
55	genistin	10 µM	1.162	0.116	0.689	0.427	0.806	0.032	0.895	0.099	1.171	0.286	1.141	0.040	0.867	0.018
		50 µM	1.025	0.097	0.508	0.248	1.078	0.017	1.393	0.217	1.790	0.245	1.163	0.236	1.027	0.058
56	prunetin	10 µM	1.164	0.165	1.401	0.411	2.132	0.164	0.740	0.281	1.372	0.243	3.044	0.216	6.138	0.204
		50 µM	0.634	0.145	1.499	0.221	1.184	0.071	0.402	0.201	1.352	0.485	0.885	0.023	10.521	1.090
57	biochanin A	10 µM	1.191	0.100	2.022	0.555	2.863	0.106	3.273	0.650	3.391	0.380	3.343	0.234	3.455	0.060
		50 µM	0.955	0.055	3.581	0.454	3.160	0.373	3.358	0.449	4.298	0.671	2.646	0.336	5.352	0.468
58	puerarin	10 µM	1.063	0.068	0.423	0.263	0.934	0.116	1.098	0.079	1.743	0.475	1.189	0.138	0.782	0.019
		50 µM	0.988	0.068	0.765	0.566	1.010	0.044	1.293	0.495	1.842	0.286	1.180	0.058	1.026	0.069
59	pelargonin	10 µM	1.030	0.066	0.678	0.287	0.988	0.102	1.396	0.116	1.310	0.410	1.158	0.111	1.058	0.110
		50 µM	0.997	0.150	0.534	0.193	0.892	0.045	1.126	0.459	1.239	0.426	0.944	0.304	1.462	0.056
60	cyanidin	10 µM	1.083	0.139	0.439	0.134	0.851	0.169	0.912	0.315	0.779	0.117	0.973	0.118	1.046	0.085
		50 µM	0.933	0.118	0.991	0.156	0.847	0.060	0.633	0.706	0.594	0.218	0.851	0.045	1.214	0.192
61	keracyanin	10 µM	0.988	0.063	1.700	0.179	0.942	0.182	0.888	0.333	1.293	0.498	1.012	0.032	0.827	0.125
		50 µM	1.015	0.083	1.493	0.527	0.893	0.159	1.295	0.489	1.336	0.478	1.149	0.027	0.796	0.062
62	cyanin	10 µM	1.167	0.124	0.390	0.247	0.950	0.087	0.873	0.273	1.035	0.043	1.137	0.129	0.719	0.090
		50 µM	1.136	0.091	1.988	1.835	0.855	0.031	1.245	0.108	1.182	0.241	1.202	0.120	0.757	0.056
63	delphinidin	10 µM	1.126	0.099	0.824	0.726	0.927	0.016	0.996	0.443	1.164	0.104	1.039	0.057	0.939	0.096
		50 µM	1.225	0.136	0.911	0.135	0.871	0.046	0.451	0.067	1.267	0.079	0.917	0.135	1.147	0.009
64	oenin	10 µM	1.167	0.091	1.136	0.752	0.934	0.205	0.877	0.235	0.989	0.342	1.086	0.145	0.852	0.087
		50 µM	1.165	0.148	1.165	1.415	0.892	0.048	0.568	0.219	1.101	0.419	0.985	0.080	1.163	0.056

Table S9. Viability (GFP expression) and homodimer STAT1 transcriptional activation (luciferase activity) in BV-2 microglia treated with combinations of 50 μM flavonoids and manganese (II) or interferon-γ for 12 hours. For “none”, n=18; for specific flavonoids, n=3.

		100 μM Mn(II)				200 μM Mn(II)				1 ng/ mL IFNγ			
		GFP fluoresc.		Luciferase act.		GFP fluoresc.		Luciferase act.		GFP fluoresc.		Luciferase act.	
Code #	flavonoid name	Fold	SD	Fold	SD	Fold	SD	Fold	SD	Fold	SD	Fold	SD
	none	0.752	0.120	5.653	0.660	0.553	0.160	4.121	0.482	0.984	0.140	51.126	4.794
1	catechin	0.856	0.050	4.083	0.607	0.792	0.060	1.701	0.464	1.301	0.153	56.213	2.005
2	epicatechin	0.988	0.080	3.669	0.601	0.948	0.089	1.377	0.229	1.345	0.112	53.948	1.049
3	ECG	0.927	0.194	2.152	0.518	0.929	0.102	0.238	0.211	1.129	0.052	40.778	1.933
4	GC	0.957	0.074	4.063	0.339	0.981	0.067	1.973	0.311	1.254	0.152	57.565	4.690
5	EGC	0.872	0.087	3.863	0.797	0.830	0.149	1.724	0.213	1.220	0.165	53.450	3.240
6	EGCG	0.721	0.021	2.269	0.225	0.720	0.121	0.234	0.198	1.181	0.092	42.559	2.960
7	naringenin	0.637	0.068	8.323	1.433	0.612	0.102	3.805	0.347	1.014	0.089	110.399	6.620
8	naringin	0.754	0.032	7.518	0.990	0.751	0.054	3.713	0.356	1.184	0.046	57.920	6.245
9	eriodictyol	0.819	0.091	2.872	0.598	0.805	0.062	0.848	0.273	0.980	0.056	53.164	5.187
10	hesperetin	0.890	0.138	16.866	1.360	0.848	0.066	10.580	1.821	1.141	0.069	158.013	8.512
11	hesperidin	0.799	0.040	4.576	0.995	0.835	0.079	2.494	0.284	1.053	0.066	53.248	3.548
12	taxifolin	0.844	0.121	3.691	0.314	0.833	0.114	1.925	0.157	1.204	0.057	54.292	2.499
13	silybin=silibinin	0.770	0.080	6.379	0.575	0.760	0.087	4.020	0.448	1.148	0.090	60.306	3.676
14	flavone	0.668	0.073	12.363	2.586	0.693	0.181	7.220	3.007	0.856	0.111	209.051	29.970
15	α-naphthoflavone	0.355	0.020	0.859	0.409	0.440	0.046	0.029	0.248	0.299	0.011	39.255	1.668
16	7-OMe-flavone	0.772	0.057	6.550	1.339	0.875	0.029	4.607	0.953	0.961	0.038	202.249	18.141
17	3’,4’-(OH)2-flavone	1.005	0.144	0.460	0.087	1.183	0.082	0.089	0.045	0.809	0.035	79.822	3.731
18	4’,5-(OH)2-flavone	0.861	0.110	2.128	0.638	0.910	0.074	1.010	0.117	0.896	0.085	166.653	19.632
19	4’,7-(OMe)2-flavone	0.578	0.136	5.572	0.885	0.631	0.135	3.151	0.710	0.554	0.073	75.587	2.292
20	5,6-(OH)2-flavone	0.665	0.033	3.520	0.811	0.786	0.167	1.280	0.185	0.837	0.049	117.435	5.645
21	chrysin	0.668	0.092	6.261	1.043	0.638	0.163	3.029	1.032	0.993	0.127	186.701	49.286
22	7,8-(OH)2-flavone	0.668	0.084	0.759	0.144	0.604	0.114	0.660	0.217	0.641	0.055	70.261	3.137
23	2',3',6-(OH)3-flavone	0.861	0.013	3.228	0.330	0.773	0.044	1.268	0.318	0.839	0.092	76.881	5.445
24	3',7,8-(OH)3-flavone	0.877	0.038	2.704	0.178	0.817	0.069	1.374	0.206	1.032	0.106	47.496	3.666
25	apigenin	0.743	0.118	0.583	0.081	0.771	0.041	0.599	0.147	0.929	0.092	129.081	8.511
26	acacetin	0.708	0.073	1.678	0.256	0.682	0.041	1.267	0.434	0.942	0.064	141.409	6.379
27	4',7,8-(OH)3-flavone	0.929	0.040	4.238	0.571	0.788	0.127	2.234	0.413	1.097	0.188	54.973	7.331
28	baicalein	0.897	0.006	4.069	0.375	0.823	0.104	2.760	0.738	1.136	0.200	75.723	5.058
29	baicalin	0.748	0.013	5.282	1.352	0.683	0.109	1.323	0.232	0.987	0.053	88.439	4.596

30	wogonin	0.540	0.056	5.445	1.401	0.353	0.042	3.676	0.511	0.848	0.028	104.378	10.153
31	luteolin	0.732	0.086	3.181	0.102	0.635	0.101	1.580	0.396	0.869	0.052	100.277	6.166
32	diosmin	0.896	0.056	4.464	0.094	0.729	0.055	2.385	0.684	0.989	0.156	45.931	0.888
33	neodiosmin	0.813	0.122	5.841	0.841	0.732	0.020	3.107	0.514	0.983	0.011	56.763	5.346
34	vitexin	1.000	0.157	6.147	1.304	0.844	0.109	4.624	0.490	1.025	0.100	62.083	4.507
35	tangeretin	0.741	0.059	4.984	1.312	0.757	0.162	3.868	0.375	0.859	0.062	92.284	5.722
36	flavonol	0.210	0.046	0.092	0.019	0.318	0.136	0.066	0.023	0.413	0.096	27.111	5.979
37	4’-OMe-flavonol	0.655	0.069	5.889	1.269	0.494	0.100	1.967	0.920	0.917	0.034	90.428	2.355
38	galangin	0.483	0.051	7.843	0.434	0.396	0.107	2.171	0.145	0.728	0.103	140.279	18.195
39	fisetin	0.686	0.051	4.444	0.959	0.608	0.081	0.917	0.086	1.050	0.074	63.036	2.637
40	kaempferol	0.707	0.075	5.447	0.189	0.642	0.061	1.634	0.476	1.086	0.079	132.285	14.222
41	4',7,8-(OH)3-flavonol	1.719	0.205	2.768	0.294	1.635	0.077	1.543	0.061	2.001	0.085	30.898	2.984
42	morin	1.038	0.088	5.102	0.723	0.835	0.040	3.479	0.732	1.139	0.095	70.772	5.389
43	quercetin	0.832	0.146	4.279	0.783	0.676	0.093	1.323	0.168	1.052	0.059	49.917	4.857
44	rhamnetin	0.669	0.025	3.970	0.463	0.600	0.112	1.298	0.307	0.988	0.154	50.460	10.360
45	quercetin-3-glucoside	0.734	0.031	3.684	0.365	0.632	0.136	0.851	0.274	0.853	0.031	51.159	2.807
46	quercitrin	0.751	0.104	4.818	0.349	0.637	0.171	2.580	0.592	1.139	0.082	50.687	3.997
47	rutin	1.028	0.178	5.252	0.829	0.767	0.124	2.491	0.421	1.288	0.179	46.909	1.806
48	myricetin	0.759	0.113	3.778	2.080	0.585	0.119	0.208	0.091	1.171	0.159	38.677	4.097
49	myricitrin	0.765	0.140	5.207	0.286	0.617	0.132	3.098	0.376	1.204	0.153	55.185	3.925
50	daidzein	0.702	0.206	15.557	2.172	0.505	0.260	5.068	3.281	1.194	0.218	205.901	12.637
51	daidzin	0.547	0.063	6.674	1.854	0.405	0.062	4.074	0.048	1.046	0.080	57.214	5.224
52	formononetin	0.829	0.122	22.732	2.277	0.674	0.151	16.432	1.198	1.189	0.097	200.346	11.406
53	3',4',7-(OH)3-isoflavone	0.835	0.094	2.458	0.271	0.591	0.091	0.919	0.342	0.949	0.164	92.508	11.095
54	genistein	0.630	0.117	6.138	1.303	0.505	0.199	3.852	0.788	1.113	0.133	175.713	12.662
55	genistin	0.725	0.108	5.540	1.157	0.522	0.183	4.105	1.311	1.151	0.166	64.532	5.262
56	prunetin	0.591	0.217	1.654	0.787	0.628	0.239	0.979	0.558	0.687	0.117	80.473	11.042
57	biochanin A	0.450	0.044	6.127	1.804	0.417	0.082	5.572	1.635	0.922	0.106	199.312	27.926
58	puerarin	0.901	0.016	6.389	0.347	0.651	0.046	5.195	0.215	0.965	0.007	54.700	1.266
59	pelargonin	0.878	0.028	7.369	0.195	0.642	0.008	4.555	0.241	0.988	0.030	53.825	1.835
60	cyanidin	0.891	0.031	6.453	0.270	0.614	0.058	4.842	0.294	0.967	0.010	56.344	2.008
61	keracyanin	0.816	0.073	6.479	0.142	0.591	0.033	4.937	0.397	0.962	0.027	56.197	1.830
62	cyanin	0.839	0.049	5.414	0.448	0.595	0.052	5.515	0.031	0.952	0.045	54.366	3.325
63	delphinidin	0.851	0.058	5.056	0.250	0.611	0.052	4.073	0.397	0.904	0.087	56.223	4.288
64	oenin	0.861	0.073	5.093	0.530	0.614	0.043	4.144	0.831	0.869	0.187	60.082	2.239

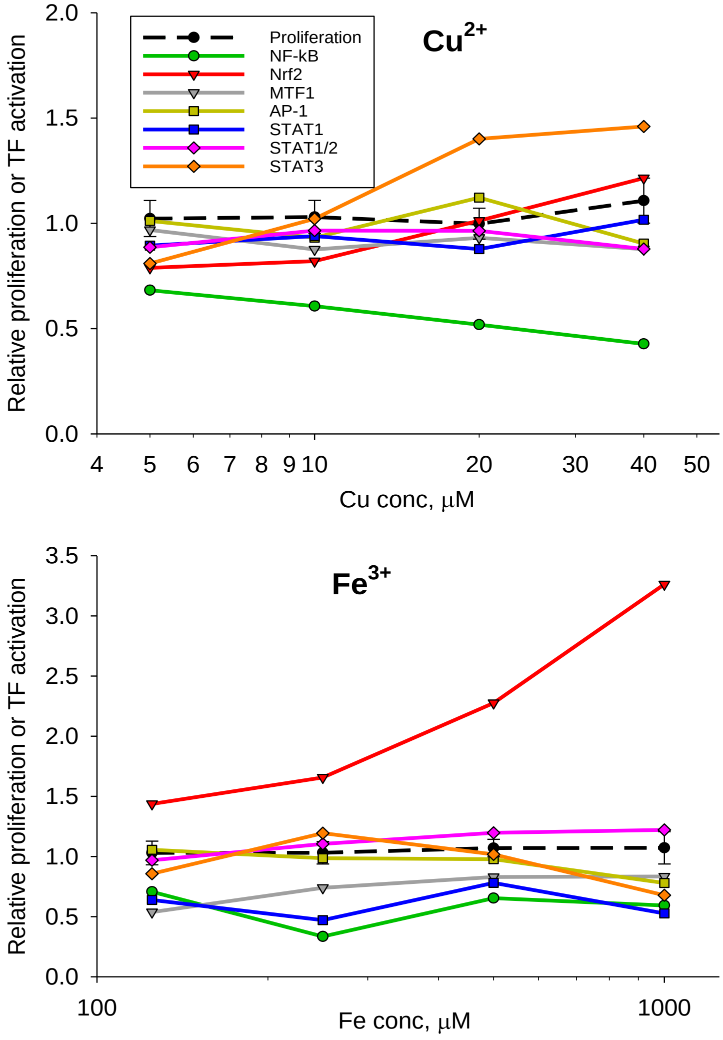

Figure S1. Proliferation rates and transcriptional responses of BV-2 microglia reporter cells treated with copper (II) chloride or iron (III) citrate for 14 hours. Color coding is the same for both panels. For proliferation rates, error bars are SDs, n = 7. For TF activation folds, n = 1.

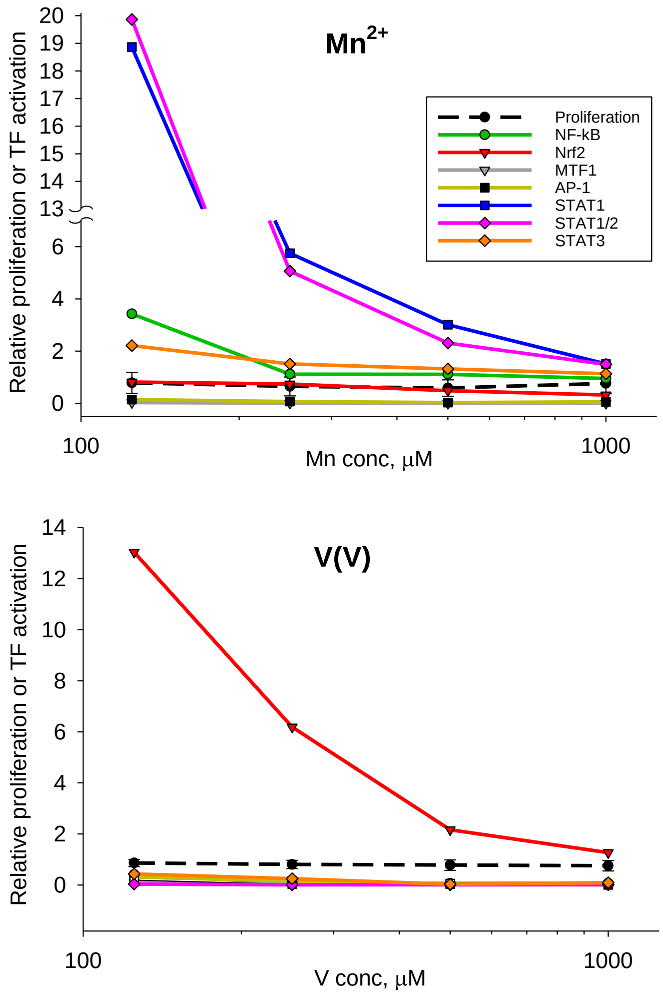

Figure S2. Proliferation rates and transcriptional responses of BV-2 microglia reporter cells treated with manganese (II) chloride or sodium orthovanadate for 14 hours. Color coding is the same for both panels. For proliferation rates, error bars are SDs, n = 7. For TF activation folds, n = 1.

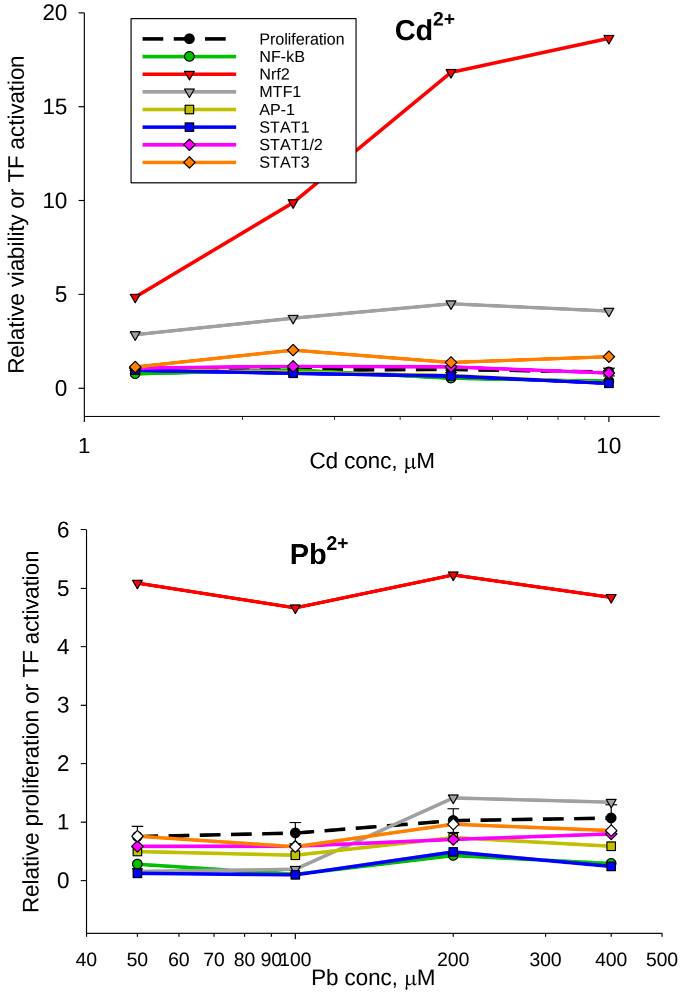

Figure S3. Proliferation rates and transcriptional responses of BV-2 microglia reporter cells treated with cadmium chloride or lead (II) acetate for 14 hours. Color coding is the same for both panels. For proliferation rates, error bars are SDs, n = 7. For TF activation folds, n = 1.

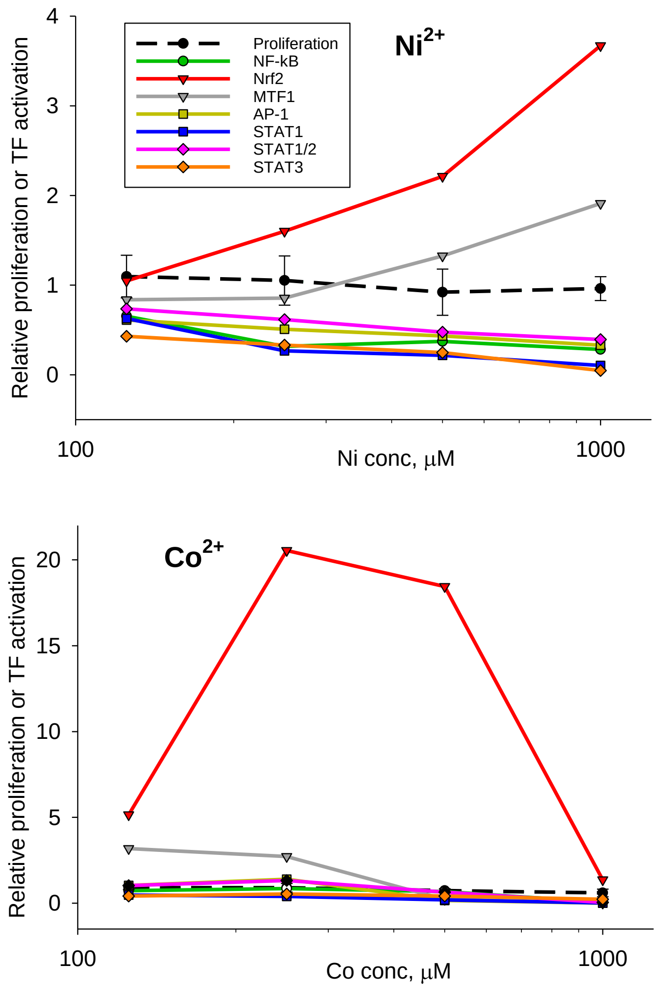

Figure S4. Proliferation rates and transcriptional responses of BV-2 microglia reporter cells treated with nickel chloride or cobalt (II) chloride for 14 hours. Color coding is the same for both panels. For proliferation rates, error bars are SDs, n = 7. For TF activation folds, n = 1.

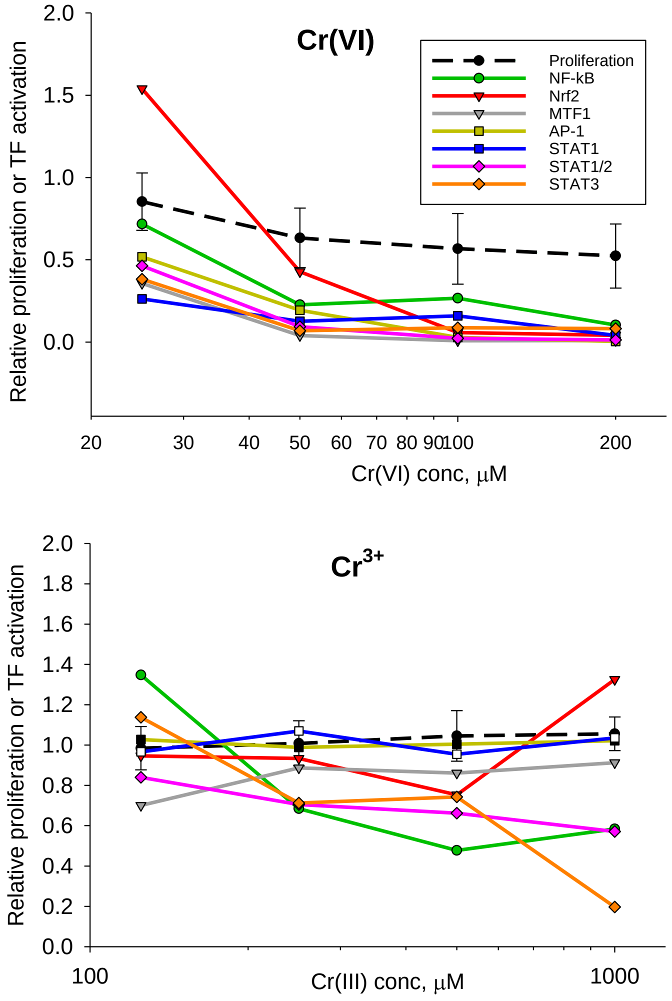

Figure S5. Proliferation rates and transcriptional responses of BV-2 microglia reporter cells treated with potassium chromate or chromium (III) chloride for 14 hours. Color coding is the same for both panels. For proliferation rates, error bars are SDs, n = 7. For TF activation folds, n = 1.

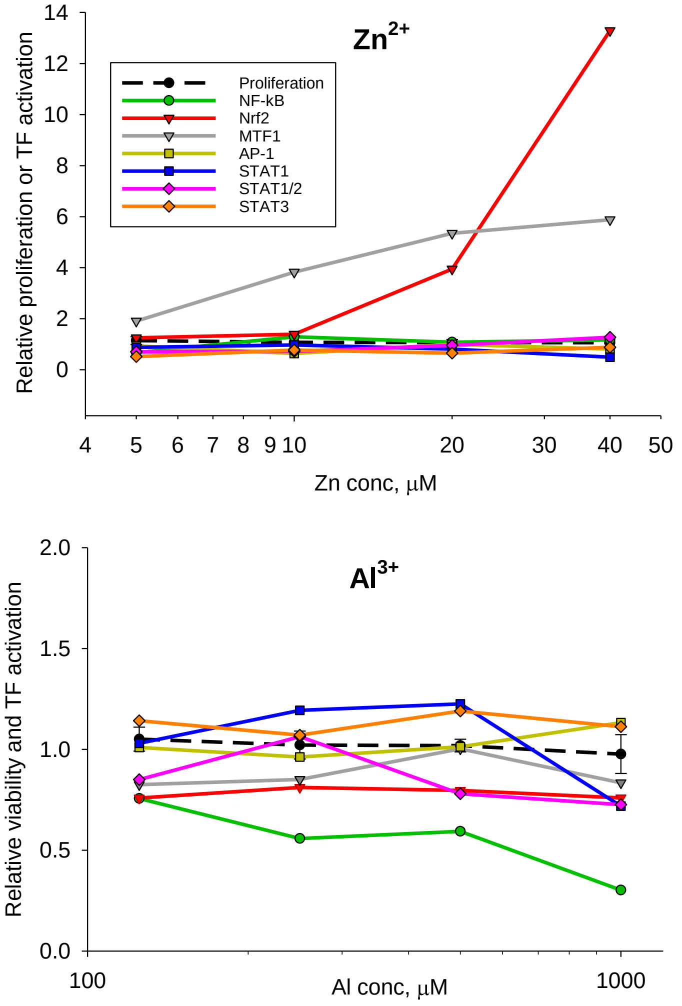

Figure S6. Proliferation rates and transcriptional responses of BV-2 microglia reporter cells treated with zinc chloride or aluminum chloride for 14 hours. Color coding is the same for both panels. For proliferation rates, error bars are SDs, n = 7. For TF activation folds, n = 1.

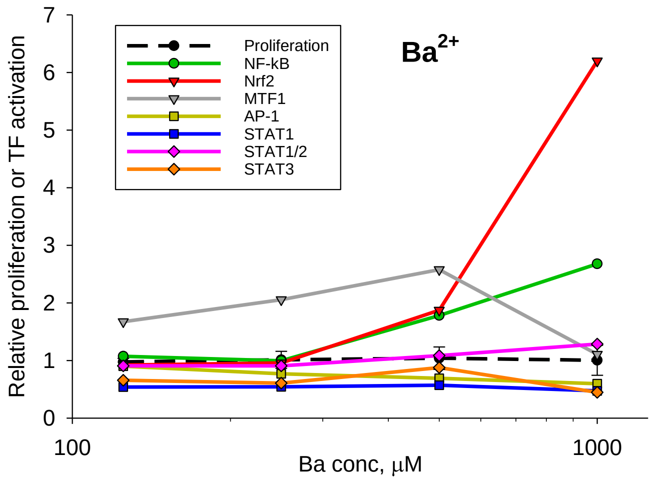

Figure S7. Proliferation rates and transcriptional responses of BV-2 microglia reporter cells treated with barium chloride for 14 hours. For proliferation rates, error bars are SDs, n = 7. For TF activation folds, n = 1.
